# Ultrasound‐Boosted Liposomal Prodrug Overcomes Age‐Associated Biodistribution Disparity in Pediatric Solid Tumor Therapy

**DOI:** 10.1002/advs.202513148

**Published:** 2025-12-27

**Authors:** Danfei Chen, Junjun Xu, Jian Chen, Jue Hu, Xiaobo Xuan, Haifang Cai, Mingdong Yang, Zhuxian Zhou, Guowei Wang

**Affiliations:** ^1^ Department of Pediatrics The First Affiliated Hospital of Zhejiang Chinese Medical University (Zhejiang Provincial Hospital of Chinese Medicine) Zhejiang Chinese Medical University Hangzhou China; ^2^ Department of Pharmacy The Second Affiliated Hospital of Zhejiang University School of Medicine Zhejiang University Hangzhou China; ^3^ College of Chemical and Biological Engineering Zhejiang Key Laboratory of Smart Biomaterials and Key Laboratory of Biomass Chemical Engineering of Ministry of Education Zhejiang University Hangzhou China; ^4^ Department of Ultrasound in Medicine The Second Affiliated Hospital of Zhejiang University School of Medicine Zhejiang University Hangzhou China

**Keywords:** age‐associated disparity, cancer‐targeting therapy, childhood solid tumor, liposomal prodrug, ultrasound

## Abstract

Clinical adoption of nanodrugs for pediatric cancer is hindered by age‐associated disparities, with unclear mechanisms limiting nanotherapeutics for childhood solid tumors. Here, we originally find that the in vivo biodistribution of nanocarriers shows marked age differences: juvenile tumor‐bearing mice have worse off‐target distribution than adults, due to vigorous neovascular proliferation in normal tissues and heightened macrophage phagocytosis in liver and spleen. To address this challenge, an ultrasound‐activated liposomal prodrug (CSCPTL) is designed to overcome age‐related disparities in off‐target distribution. CSCPTL is composed of a camptothecin‐lipid prodrug conjugate (SCPT) containing the reactive oxygen species (ROS)‐cleavable thioketal linker, a sonosensitizer (chlorin e6, Ce6)‐modified lipid, and other commercially available lipids. Upon intravenous injection, the inactive SCPT exerts minimal pharmacological effects on healthy cells. Conversely, once CSCPTL reaches the tumor site and is internalized by cancer cells, ultrasound irradiation activates Ce6 rapidly to generate massive ROS, which cleaves the thioketal linker to release active camptothecin to induce cell apoptosis. CSCPTL showed potent antitumor efficacy in juvenile hepatoblastoma models, showing superior biocompatibility and no side effects, compared with clinically approved nanodrugs such as Abraxane, Doxil, and Onivyde. This study highlights age‐related off‐target issues and offers a promising ultrasound‐controlled strategy for childhood solid tumors.

## Graphical abstract

The age‐associated disparity of nanocarrier biodistribution limits the nanotherapeutics of childhood solid tumors. This study developed an ultrasound‐activated liposomal prodrug (CSCPTL) to overcome the age‐associated disparity of off‐target distribution. CSCPTL can merely be activated under ultrasound irradiation in combination with sonosensitizer to achieve potent anticancer activity in multiple childhood hepatoblastoma‐bearing mice while endowing better biocompatibility with no chemotherapy‐induced side effects.

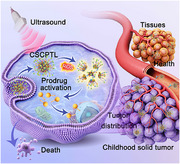

## 1 Introduction

Pediatric malignancies are the leading cause of non‐accidental death among children worldwide [1, 2, 3]. An estimated 400 000 children and adolescents (under 18 years of age) are diagnosed with cancer annually, with approximately 100 000 deaths each year [3, 4, 5]. Alarmingly, both incidence and mortality rates are increasing. Current treatment regimens for pediatric cancers primarily depend on cytotoxic agents, most of which were originally developed for adult cancers. Only a limited number have been formally approved for pediatric use, often through dosage adjustments or off‐label applications [3, 6]. In addition, many pediatric cancers, especially childhood solid tumors, pose particular treatment challenges [6]. Even in cases of successful control, standard pharmacotherapies can lead to long‐term adverse effects that significantly impact survivors' physical and psychosocial development, therefore innovative, targeted, and safer therapeutic strategies are urgently required for pediatric malignancies worldwide.

Nanomedicine has shown significant promise in improving cancer diagnosis, therapy, monitoring, and control compared with conventional cytotoxic agents. Over the past three decades, extensive research into various nanotherapeutics (e.g., protein‐drug conjugates, liposomes, micelles, polymers, dendrimers, hydrogels, and inorganic nanoparticles) has led to numerous commercial nanomedical products and a growing clinical pipeline of candidates for cancer diagnosis and treatment [7, 8, 9, 10, 11]. Despite these advances, the clinical translation of nanotherapeutics for pediatric solid tumors remains limited by age‐associated disparities [12, 13, 14]. Pediatric cancers exhibit distinct epidemiologic, genetic, clinical, pathological, and physiological profiles, requiring tailored treatment approaches. A significant barrier is the lack of pediatric representation in clinical trials, particularly those addressing adult‐pediatric co‐morbidities [15]. This contributes to a limited understanding of nanodrug design and dosing for pediatric populations. Addressing these gaps is essential to achieve safe and effective nanodrug options and improve outcomes for children with solid tumors.

Compared with adult patients, children's distinct physiological characteristics, such as rapid organ development, active neovascularization networks, and an immature immune system, contribute to heightened immunogenicity and inflammatory responses, particularly in the liver and spleen [6, 13]. These characteristics may increase the likelihood of off‐target nanocarrier accumulation, significantly reduce pediatric patient compliance, and lead to poor prognosis in long‐term growth. For instance, liposomal doxorubicin (Doxil) and other anthracyclines have indeed been associated with a higher incidence of infusion reactions, palmoplantar erythrodysesthesia, and multi‐organ dysfunction syndrome in pediatric patients in clinical observations [16, 17, 18, 19]. Hence, mitigating age‐associated disparities in nanodrug biodistribution is as critical as enhancing antitumor efficacy in pediatric solid tumor treatment.

**SCHEME 1:**
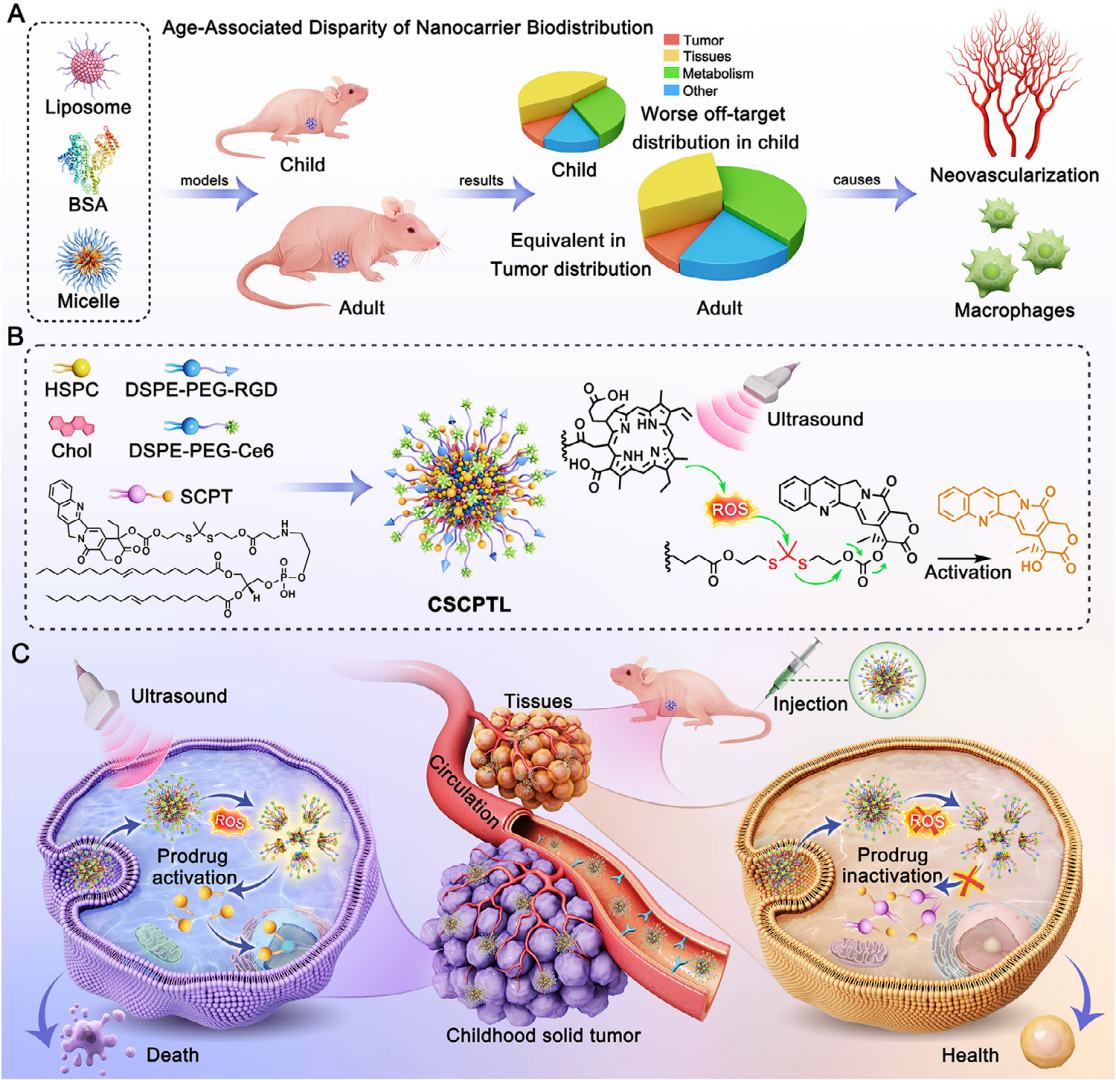
Schematic representation of the US‐activated liposomal prodrug (CSCPTL) and its delivery mechanism for nanotherapeutics of childhood solid tumors. (A) The age‐associated disparity in nanocarrier biodistribution and its underlying causes in juvenile and adult hepatoblastoma‐bearing mice. (B) Composition and fabrication of CSCPTL, including the ROS‐induced cleavage mechanism of the thioketal linker upon US activation. (C) The delivery mechanism of CSCPTL in pediatric solid tumor therapy. After intravenous injection, although some CSCPTL inevitably accumulates in the normal tissues of juvenile tumor‐bearing mice, the inactive SCPT exerts minimal pharmacological activity. By contrast, once CSCPTL is internalized into tumor cells, US irradiation activates the sonosensitizer Ce6, rapidly generating ROS. This initiates ROS‐mediated cleavage of the thioketal linker, releasing active CPT to induce apoptosis in cancer cells.

Herein, we first evaluated age‐associated disparities in the in vivo biodistribution of nanocarriers using hepatoblastoma models in both juvenile and adult mice (Scheme 1A). It is revealed that the off‐target distribution of the nanocarrier is markedly worse in juvenile mice, potentially leading to more severe side effects. Current nanodrugs are highly dependent on tumor neovascularization and suffer from an Achilles heel of significant off‐target accumulation in the liver and spleen [20, 21]. Meanwhile, tumor microenvironment‐responsive nanodrugs frequently show limited efficacy, primarily due to significant inter‐ and intra‐tumor heterogeneity across different patient and tumor types [8, 9, 22]. Given that off‐target distribution of nanodrugs in pediatric patients appears unavoidable, we propose an ultrasound (US)‐activated liposomal prodrug (CSCPTL) that remains pharmacologically inactive in normal tissues but is only activated under exogenous stimuli of US within tumors. The CSCPTL is composed of the camptothecin (CPT)‐dioleoyl phosphatidylethanolamine prodrug conjugate (SCPT) containing a ROS‐sensitive thioketal linker, Ce6‐modified PEG2000‐DSPE, and other commercial lipids (Scheme 1B). Under US exposure, Ce6 induces robust ROS production, disrupting the intracellular endogenous oxidation‐reduction chemical equilibrium to trigger oxidation‐sensitive reactions in a non‐intrusive, homogeneous manner, as proven and used in our previous work [23, 24]. Following intravenous injection in juvenile tumor‐bearing mice, even if some of the CSCPTL is inevitably distributed in normal tissues, the inactive SCPT exerts minimal pharmacological activity on normal cells. Conversely, upon tumor accumulation and cellular uptake, under US irradiation, Ce6 rapidly induces massive ROS production, after initiating a ROS‐responsive CSCPTL activation to release the active CPT and thus induce cell apoptosis (Scheme 1C). This study might offer a promising new direction in pediatric nanomedicine—one that overcomes the age‐associated disparity of nanocarrier biodistribution to reduce off‐target effects and enhance therapeutic precision for childhood solid tumors.

## 2 Results and Discussion

### 2.1 Establishment of Childhood Solid Tumor and Nanocarrier Models, and In Vivo Biodistribution

**FIGURE 1:**
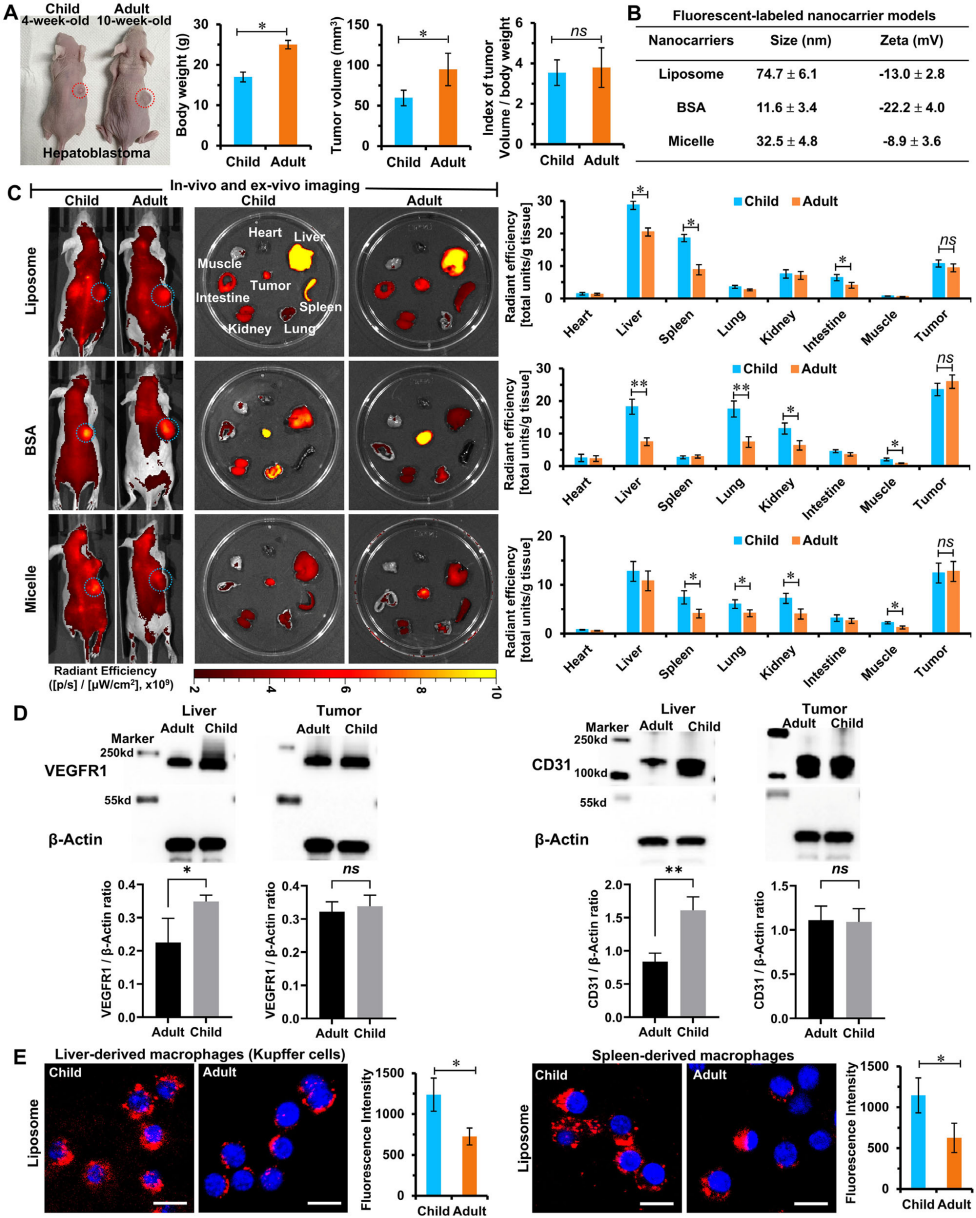
In vivo biodistribution of nanocarriers in childhood and adulthood solid tumor models, and the evaluation of neovascularization and macrophages. (A) The photographs of representative mice from childhood and adulthood, solid tumor models, and their tumor volume and body weight analysis (n = 10). (B) The particle size and zeta potentials of the three selected typical fluorescent‐labeled nanocarrier models: liposome, BSA, and micelle (n = 3). (C) The in vivo and *ex vivo* fluorescent imaging of mice and tissues at 24 h after an intravenous injection of nanocarriers. All images were obtained using the Caliper IVIS Lumina II system (PerkinElmer), and the fluorescence intensity of tissues’ distribution was quantitatively analyzed by Living Image‐4.5 software in terms of the absolute intensity of total units/tissue weight (n = 3). (D) The Western blotting of neovascularization in childhood and adulthood solid tumor models in terms of the typically expressed biomarkers: VEGFR1 and CD31 (n = 3). (E) The phagocytosis evaluation of macrophages derived from the liver and spleen of childhood and adulthood solid tumor models. The macrophages were visualized using CLSM after incubation with the liposome model for 0.5 h (scale bar = 50 µm), and the fluorescence intensity of macrophages was collected and quantified by flow cytometry (n = 4). Data were expressed as the mean ± SD, and significances were determined by unpaired two‐tailed Student's *t*‐test or one‐way ANOVA, ^*^ *p* < 0.05, ^**^ *p* < 0.01, and *ns* means no significant difference.

To explore age‐associated disparities in nanocarrier biodistribution, solid tumor models were established in 4‐week‐old (child) and 10‐week‐old (adult) HepG2 tumor‐bearing mice (Figure 1A), a model of hepatoblastoma—a malignancy with high prevalence in both pediatric and adult populations [25, 26, 27]. While the adult mice exhibited significantly greater body weights and tumor volumes than juvenile mice, the tumor volume‐to‐body weight ratio remained consistent across groups. This ensured comparable systemic drug exposure per mouse and accurate tissue‐level dosing based on weight‐adjusted conversions. Three representative fluorescently labeled nanocarrier systems: liposomes, bovine serum albumin (BSA) nanoparticles, and micelles, were prepared for biodistribution analysis.

Liposome model (size: 74.7 ± 6.1 nm; zeta potential: −13.0 ± 2.8 mV) was formulated using hydrogenated soybean phosphatidylcholine (HSPC), cholesterol, 1,2‐distearoyl‐sn‐glycero‐3‐phosphoethanolamine‐N‐[methoxy (polyethyleneglycol)‐2000] (PEG2000‐DSPE), and Cy5‐PEG2000‐DSPE in a molar ratio of 56.5:38.2:5.3:0.1, following a clinically validated Doxil‐inspired formulation [28]. BSA nanoparticles (size: 11.6 ± 3.4 nm; zeta potential: −22.2 ± 4.0 mV) were synthesized from Cy5‐conjugated BSA (molecular weight: 66.5 kDa). Micelles (size: 32.5 ± 4.8 nm; zeta potential: −8.9 ± 3.6 mV) were fabricated using Cy5‐conjugated poly (ethylene glycol 2000)‐polystyrene 2000 block copolymers (Figure 1B; Figure S1). All nanocarriers were well‐defined nanospheres with excellent dispersity (polydispersity index ≤ 0.2) and demonstrated high stability, ensuring reliable evaluation of in vivo biodistribution.

Nanocarrier biodistribution was assessed at 24 h after intravenous injection by using the Caliper IVIS Lumina II system (PerkinElmer), and tissue‐specific fluorescence was quantified using Living Image‐4.5 software as the absolute intensity of total signal per tissue weight (Figure 1C). Across all three nanocarrier types, no significant differences in tumor fluorescence intensities were observed between juvenile and adult models, suggesting that the nanodrugs have similar tumor‐targeting efficiencies and potential therapeutic efficacy in both age groups. However, significant differences emerged in the accumulation of nanocarriers in normal tissues. In liposome‐treated mice, the liver and spleen exhibited the highest off‐target fluorescence. In the BSA groups, substantial accumulation was observed in the liver, lungs, and kidneys, albeit less than in tumors. In micelle‐treated mice, fluorescence in the liver, spleen, lungs, and kidneys exceeded that in tumors. Critically, all in vivo biodistribution of nanocarriers exhibited an age‐associated disparity: fluorescence accumulation of nanocarriers in normal tissues was significantly higher in juvenile mouse tumor models than in adult mouse tumor models (e.g., liver, spleen, and intestine in liposomes; liver, lung, kidney, and muscle in BSA nanoparticles; and spleen, lung, kidney, and muscle in micelles). Despite their distinct physical, chemical, and biological characteristics, all nanocarriers showed a consistent trend of exacerbated off‐target distribution in juvenile models, highlighting a major safety concern related to pediatric use of nanodrugs.

The reasons resulting in age‐associated disparity of nanocarrier biodistribution were further investigated. During transportation following nanocarrier intravenous injection, two key physiological factors critically influencing nanocarrier biodistribution are neovascularization and macrophage phagocytosis [20, 29, 30, 31]. Neovascularization directly affects nanocarrier delivery via the vascular supply to both tumors and normal tissues. Macrophage phagocytic activity directly governs nanoparticle uptake, especially in the liver and spleen, which are primary sites of the mononuclear phagocyte system. Western blot analysis of neovascularization markers—vascular endothelial growth factor receptor‐1 (VEGFR1) and platelet endothelial cell adhesion molecule‐1 (CD31)—revealed no significant difference between juvenile and adult tumors (Figure 1D; Figure S2). However, their protein expression levels were significantly higher in normal tissues (e.g., liver, lung, kidney, muscle) of juvenile mice than in adult mice, indicating enhanced neovascular development of normal tissues in juvenile tumor‐bearing mice. The extensive neovascularization offers the normal tissues with abundant nutrient supply, as well as inevitably transports considerably more nanocarriers to normal tissues, resulting in elevated off‐target accumulation. Macrophages were isolated from the liver and spleen by using adherence screening and identified using Trypan Blue staining (Figure S3). Viable macrophages absorbed the dye and quickly adhered to the culture dish, while other cell types and nonviable macrophages were removed using the cell culture medium. These macrophages were incubated with liposomes and analyzed through confocal laser scanning microscopy and flow cytometry after 0.5 h incubation (Figure 1E). Macrophages derived from juvenile mice (liver or spleen) displayed higher fluorescence intensity and nanocarrier uptake than those from adult mice, indicating elevated phagocytic activity. This is consistent with previous findings showing increased expression of the macrophage scavenger receptor MARCO—known to mediate phagocytosis of targets (e.g., nanoparticles and bacteria)—in liver‐derived macrophages of juvenile mice than of aged mice [12]. Together, these results highlight the dual impact of enhanced neovascularization and elevated macrophage phagocytosis. Besides these immediate causes, the protein corona that formed on the surface of nanomedicines in the bloodstream might be a potential cause for the age‐associated disparity of nanocarrier biodistribution. The protein components of blood between juveniles and adults are significantly different in clinical practice. The protein corona can critically alter nanomedicines' physicochemical properties, influence their interactions with biosystems, and affect pharmacokinetics and biological outcomes. These age‐associated physiological features contribute to increased off‐target nanodrug distribution in children with cancer patients, raising substantial safety concerns. Considering the need for long‐term growth and development in pediatric cancer survivors after nanotherapeutics, the age‐associated disparity of nanocarrier biodistribution is a challenging barrier and safety concern in nanodrug designing and clinical adoption that must be addressed.

### 2.2 Design of ROS‐responsive CPT‐lipid Prodrug Conjugates, and Fabrication of Liposomes

**FIGURE 2:**
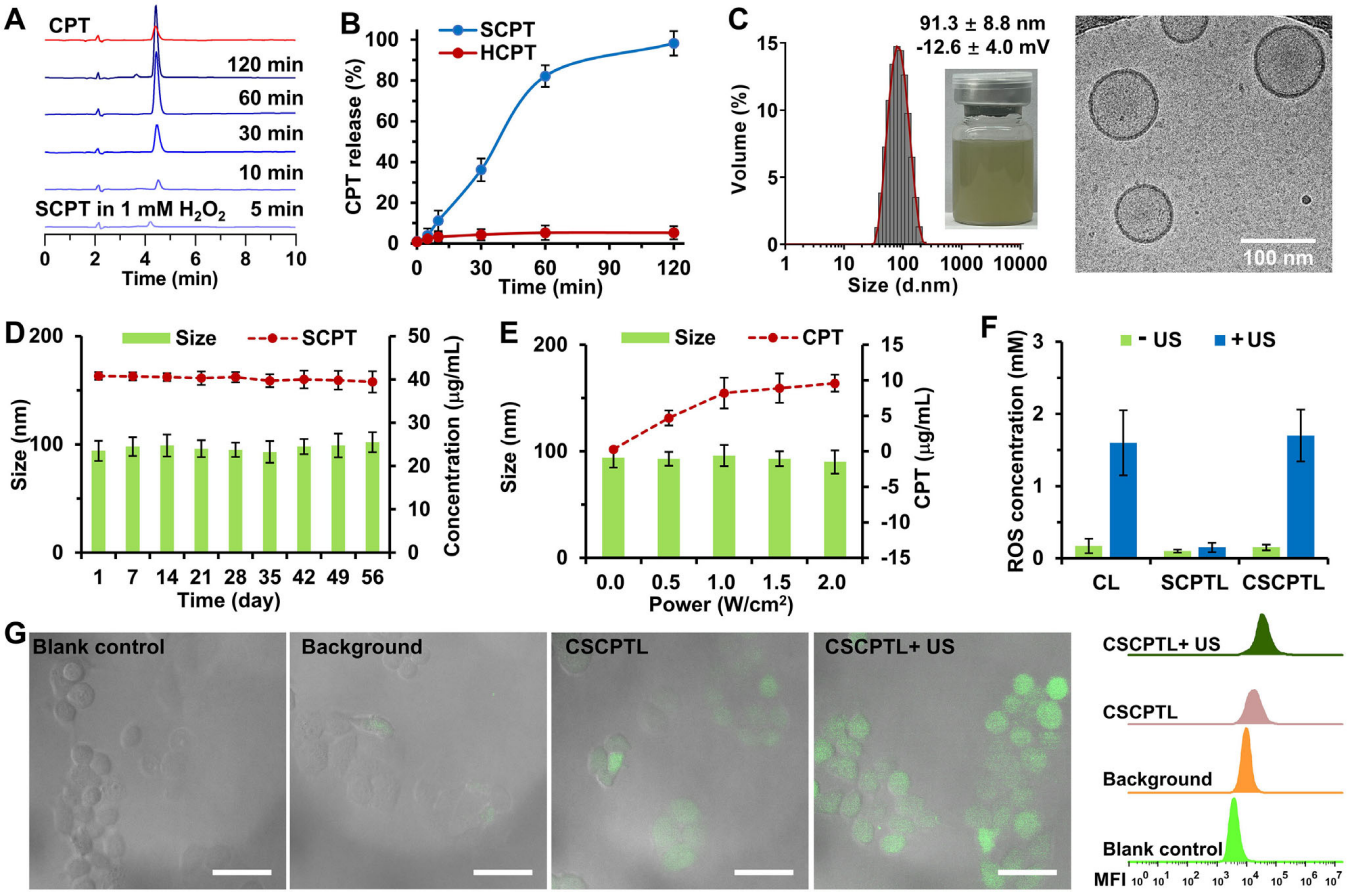
Physiochemical and biological characterizations of CPT‐lipid prodrugs and liposomes. (A) The ROS‐responsive CPT‐lipid prodrug conjugates at different incubation times in the concentrations of 1 mm H_2_O_2_, and (B) the kinetics of activated CPT release from CPT‐lipid prodrug conjugates. The conjugates were separately incubated with 1 mm H_2_O_2_ solution with the conjugate and H_2_O_2_ at a molar ratio of 1% and quantitatively analyzed by HPLC (n = 3). (C) The macroscopic morphology and size distribution were measured by dynamic light scattering and cryo‐TEM images of CSCPTL (scale bar = 100 nm). (D) Stability evaluation of CSCPTL during 8 weeks (n = 3). (E) US‐responsive drug activation and size distribution under different acoustic intensities (n = 3). (F) Quantitative results of ROS concentration in the liposome solution without or with US irradiation (n = 3). (G) CLSM images depicting the intracellular ROS production of CSCPTL without or with US irradiation, and the representative flow cytometry plots (scale bar = 50 µm). The quantitative results of the flow cytometry analysis of the intracellular ROS levels in HepG2 cells were respectively treated with blank control (adding CSCPTL liposome), background control (adding SCPTL liposome and DCFH‐DA), CSCPTL group (adding CSCPTL liposome and DCFH‐DA), and CSCPTL+US (adding CSCPTL liposome and DCFH‐DA and US irradiation). The data are presented as the mean ± SD. The US irradiation (acoustic intensity: 1 W cm^−2^, frequency: 3 MHz, duty cycle: 50%) was performed using a Mettler Sonicator‐740 equipped with a 1‐cm^2^ ultrasonic probe.

Although cancer nanomedicines have brought about thousands of breakthroughs in revolutionizing cancer treatments by offering targeted and controlled drug delivery, as well as improving patient outcomes, the off‐target distribution of nanodrugs remains a huge issue even for the targeting drug‐delivery system. As it was factually impossible to avoid off‐target distribution of nanodrugs in pediatric patients, especially the liver and spleen capture, a distinct proposal was made of designing a drug‐delivery system that could leave the off‐target nanodrugs inactive but activate the tumor‐distributed nanodrugs under exogenous stimuli in an endogenic factors‐independent manner. Selecting a universal and efficient exogenous stimulus is the key to successfully designing a drug‐delivery system. US‐responsive drug delivery has demonstrated great potential for improving prodrug activation, extravasation, and penetration in non‐intrusive, in‐depth areas in a homogeneous manner [32, 33, 34]. Ultrasonic chemical effects can induce massive ROS production under sono‐sensitization, and the newly produced ROS can break the intracellular oxidation‐reduction chemical equilibrium to trigger oxidation‐sensitive reactions [23, 24]. To realize intracellular exogenous stimulus‐responsive prodrug activation, the chemotherapeutic drug CPT was conjugated to DOPE via a ROS‐cleavable thioketal linker. The CPT‐DOPE conjugate containing thioketal linker (SCPT) was obtained and characterized in detail by ^1^H‐NMR and MALDI‐TOF‐MS (Figures S4–S6). Meanwhile, a non‐ROS‐responsive CPT‐DOPE conjugate (HCPT) was synthesized using the hexanediol linker (Figures S7 and S8). Upon incubation with 1 mm hydrogen peroxide (H_2_O_2_), SCPT was activated and released over 90% of CPT within 120 min, whereas HCPT was insensitive and only released <5% of CPT in 120 min, as monitored by high‐performance liquid chromatography (HPLC) (Figure 2A,B). These results confirmed the successful design and synthesis of the ROS‐sensitive CPT‐lipid prodrug conjugate.

To ensure the efficiency of ultrasonic chemical effects, a highly efficient US‐induced ROS producer, the sonosensitizer Ce6‐modified 1,2‐distearoyl‐sn‐glycero‐3‐phosphoethanolamine‐N‐[methoxy (polyethylene glycol)‐2000] (Ce6‐PEG2000‐DSPE) lipid was selected for liposome fabrication. The CSCPTL liposome was then prepared using a lipid mixture of SCPT, Ce6‐PEG2000‐DSPE, RGD‐PEG2000‐DSPE, HSPC, and cholesterol in a mass ratio of 2:4:1:2:1 (Table S1), in accordance with our previous report, albeit with some modifications [35, 36]. The lipoid SCPT, which is a lipid component, can fully integrate with the phospholipid membrane, yielding approximately 100% drug encapsulation efficiency and an equivalent CPT‐loading rate of approximately 5.1%. The yielded CSCPTL liposome was in the form of green‐yellow emulsion with a particle size of 91.3 ± 8.8 nm (polydispersity index: 0.16) and a zeta potential of −12.6 ± 4.0 mV in PBS buffer at 25°C, as measured by dynamic light scattering, showing a homogeneous dispersion and uniform phospholipid bilayer structure under cryo‐transmission electron microscopy (cryo‐TEM) (Figure 2C). The particle size distribution of CSCPTL in cryo‐TEM images was further analyzed using the software of Nano Measurer. The particle size distribution of CSCPTL was 90.6 ± 47.4 nm, which was in line with the result measured by dynamic light scattering (Figure S9A). Meanwhile, two control liposomes, including the non‐drug loading (i.e., without SCPT component) liposome (CL) and the non‐sonosensitizer loading (i.e., without Ce6‐PEG2000‐DSPE component) liposome (SCPTL), were prepared simultaneously (Table S1). The size, zeta potentials, and cryo‐TEM images of CL and SCPTL were similar to those of CSCPTL; moreover, SCPTL exhibited milky emulsion under macroscopic morphology (Figure S9B).

The stability of CSCPTL was evaluated under different conditions of storage time, temperature, and light exposure (Figure 2D; Figure S10). The particle size and SCPT content of CSCPTL can remain stable at 4°C for 8 weeks in the dark, and even at high temperatures (e.g., 37°C). However, with increasing luminous intensity, the light exposure could only slightly decrease the SCPT content, because the sonosensitizer Ce6 was also a type of photosensitizer for producing ROS to some extent, suggesting that CSCPTL should be stored in the dark. The US‐responsive prodrug activation and ROS production were evaluated under different acoustic intensities (Figure 2E–F; Figure S11). With the increasing acoustic intensity, the amount of SCPT transforming into CPT was enhanced in the CSCPTL group, whereas the SCPT in the SCPTL group showed no changes under US irradiation. Meanwhile, ROS concentration in the CSCPTL group was sharply increased by approximately 10 folds, while that of the SCPTL group remained unchanged. These results proved the US‐triggered ROS production for SCPT prodrug activation. To ensure a convincingly visual ROS production in cancer cells, CLSM imaging of the intracellular ROS production of CSCPTL without or with US irradiation was performed using an intracellular ROS‐sensitive fluorescent probe of 2′‐7′‐dichlorodihydrofluorescein diacetate (DCFH‐DA). CLSM images verified that DCFH‐DA fluorescence was very weak in the CSCPTL group without US irradiation. On the contrary, the green fluorescing signals were significantly luminous in the CSCPTL group with US irradiation, and the fluorescence intensity was increased by approximately 6 folds in the flow cytometry assay, confirming the considerably higher intracellular ROS production (Figure 2G; Figure S12). These results together confirmed the successful design of intracellularly exogenous stimulus‐responsive prodrug activation of CSCPTL liposome.

### 2.3 Cytotoxicity, Cellular Uptake, and Subcellular Distribution of Liposomes

**FIGURE 3:**
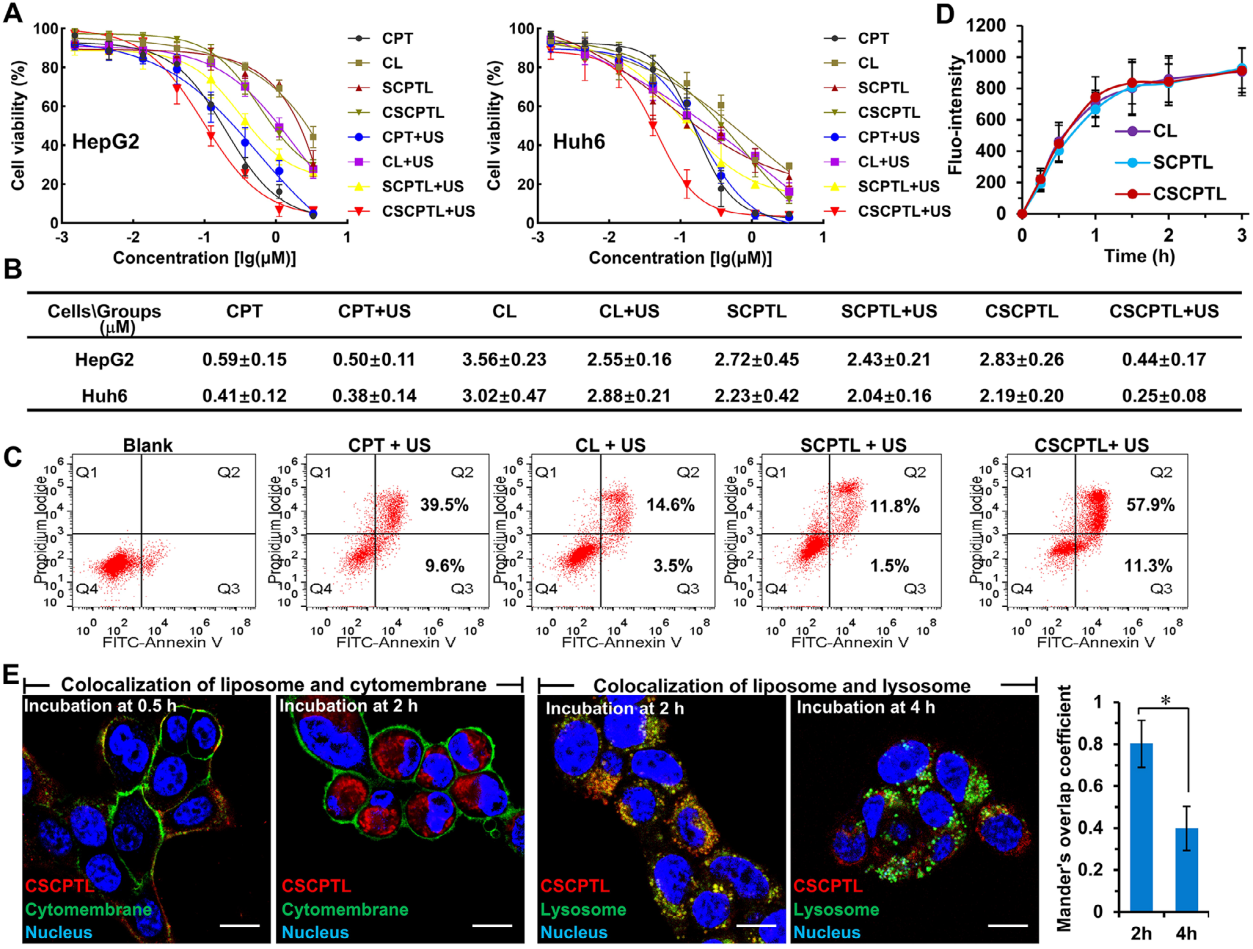
In vitro cytotoxicity, cellular uptake, and subcellular distribution of liposomes. (A) Cell viability was measured by CCK‐8 assay after treatment with free CPT and liposomes in HepG2 and Huh6 cells without or with US irradiation under different CPT‐equivalent concentrations, and (B) the IC50 values were calculated (n = 6). The cells were incubated with liposomes for 6 h, followed by US irradiation (acoustic intensity: 1 W cm^−2^, frequency: 3 MHz, duty cycle: 50%, duration: 5 min) and incubation for 42 h. (C) Representative flow cytometry of HepG2 cell apoptosis after different treatments at the CPT‐equivalent concentration was 0.5 µm (n = 3). (D) Time‐dependent cellular uptake of the liposomes in HepG2 cells (n = 3). The Cy5‐labeled liposomes were prepared by integrating the fluorescence lipid of Cy5‐PEG2000‐DSPE at 0.5% mass ratio of the total lipids. (E) The subcellular distribution of liposomes in HepG2 cells after 2 h and 4 h incubation. Nuclei (blue) were stained with Hoechst 33342, cell membranes (green) were stained with 3,3'‐dioctadecyloxacarbocyanine perchlorate (DIO), lysosomes (green) were stained with LysoTracker Green, and liposomes (red) were labeled with Cy5 (scale bar = 25 µm). The Mander's overlap coefficient of liposomes (red) with lysosomes (green) after 4 h of incubation was analyzed using an image analysis software, Cellprofiler V2.2.0 (n = 5). The data were expressed as the mean ± SD, and the significances were determined by an unpaired two‐tailed Student's *t*‐test, ^*^ *p* < 0.05.

The in vitro cytotoxicity of liposomes against human hepatoblastoma of HepG2 and Huh6 cell lines was investigated (Figure 3A,B). The free CPT and liposomal prodrugs all demonstrated dose‐dependent cytotoxicity against both cell lines without or with US irradiation. Without US treatments, liposomal prodrugs of SCPTL and CSCPTL exhibited weak cytotoxicity in comparison with free CPT and a similar tendency of cytotoxicity when compared with blank liposome of CL, thereby indicating the negligible pharmacological action of an inactivated prodrug. However, under US treatments, the cytotoxic activity of CSCPTL was sharply increased, and the IC_50_ of CSCPTL was 0.44 ± 0.17 µm against HepG2 and 0.25 ± 0.08 µm against Huh6, respectively, which was approximately 6–12 times more than CL and SCPTL liposomes. No significant differences were noted in terms of the IC_50_ values between US‐treated and non‐US‐treated subgroups in both the CPT and SCPTL groups, whereas both CL and CSCPTL groups with US treatment displayed higher cytotoxicity relative to those without US treatment, suggesting the sonodynamic therapy of sonosensitizer to some extent, apart from prodrug activation. In addition, the cytotoxicity of liposomes was evaluated on two normal cell lines of NIH/3T3 (a fibroblast cell line) and HK2 (a proximal tubular cell line) (Figure S13), which were widely used in drug toxicity assay [37]. Under no‐US treatments, all liposomes showed no cytotoxic effects on both NIH/3T3 and HK2 cells, even after increasing the CPT‐equivalent concentration to 10 µm, whereas the free CPT resulted in serious apoptosis for normal cells, indicating a better biocompatibility and safety of the liposomes. Flow cytometry was simultaneously performed to analyze cell apoptosis after Annexin‐V‐FITC/PI staining (Figure 3C; Figure S14). Under non‐US treatments, all liposomes showed negligible cytotoxic effects on HepG2 cells. On the contrary, once under US irradiation, CSCPTL displayed the highest cytotoxic activity, exhibiting an early apoptosis rate of nearly 60%, which was significantly higher than those of CPT (39.5 ± 6.3%), CL (14.6 ± 3.4%), and SCPTL (11.8 ± 2.2%). These results collectively proved that the sonosensitizer, along with US irradiation, produced intracellular ROS to trigger SCPT activation and induce cell apoptosis. This exogenous stimulus‐initiating intracellular oxidation‐responsive prodrug activation can be designed to treat solid tumors in a temporal‐ and spatial‐controllable manner while avoiding the potential damage to normal tissues.

The cellular uptake and intracellular trafficking of liposomes were then examined by confocal laser fluorescent imaging and flow cytometry. The three liposomes exhibited similar cellular uptake efficiency and could be fully internalized within 2 h (Figure 3D), indicating that the lipid components of SCPT or Ce6‐PEG2000‐DSPE had no effects on the cellular uptake of liposomes. The subcellular distribution of CSCPTL was investigated using CLSM by the colocalization of cell membranes or lysosomes (Figure 3E). CSCPTL could quickly adhere to the cellular membrane after 0.5 h of incubation and, subsequently, fully enter into the cells after 2 h of incubation, possibly through integrin receptor‐mediated endocytosis due to the RGD peptide binding to integrin αvβ3 receptor on the surface of cancer cells [38, 39]. Moreover, most CSCPTL was first distributed into the lysosomes after 2 h of incubation, albeit CSCPTL could escape from lysosomes and achieve cytoplasm distribution with extended incubation, thereby providing an opportunity for the drug's pharmacological action in the cytoplasm for targeting the enzyme topoisomerase‐1.

### 2.4 Blood Clearance, Biodistribution, In Vivo Antitumor Activity, and Biocompatibility

**FIGURE 4:**
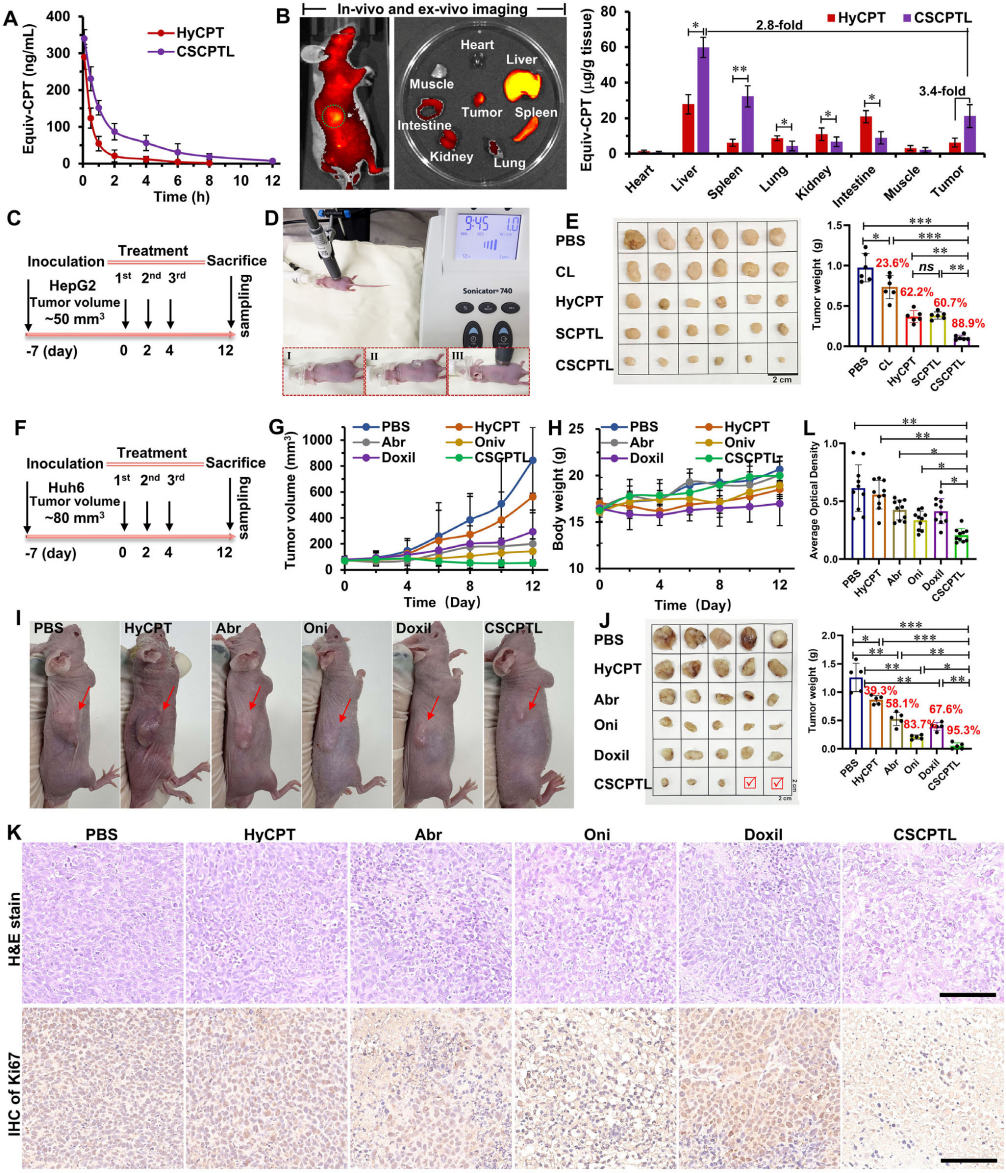
Pharmacokinetics, biodistribution, and in vivo antitumor activity on childhood BALB/c nude mice bearing hepatoblastoma. (A) The pharmacokinetics of the HyCPT and liposome were investigated in childhood tumor‐bearing mice after receiving an intravenous injection (equivalent to a CPT dose of 5 mg kg^−1^, n = 3). (B) In vivo and *ex vivo* fluorescent imaging of mice and tissues at 24‐h post‐injection. Images were obtained using the Caliper IVIS Lumina II system, and the fluorescence intensity and drug concentration of accumulated tissues were quantitatively analyzed (n = 3, unpaired two‐tailed *t*‐test, ^*^ *p* < 0.05, ^**^ *p* < 0.01). (C) The protocol of the experimental timeline and the childhood HepG2 tumor treatment schedule. (D) The US apparatus was designed for tumor treatment in this experiment. US irradiation (intensity: 1 W cm^−2^, frequency: 3 MHz, duty cycle: 50%, duration: 10 min, Mettler Sonicator‐740) was applied at 6 h after liposome injection in each mouse. (E) Photograph of the excised tumors and the average tumor weight of each group at the end of the HepG2 tumor treatment experiment (n = 6). The childhood HepG2 tumor‐bearing mice were intravenously injected with HyCPT, SCPTL, and CSCPTL (equivalent to CPT dose 5 mg kg^−1^), or blank control CL and PBS, and then treated with US irradiation (intensity: 1 W cm^−2^, frequency: 3 MHz, duty cycle: 50%, duration: 10 min) at 6h post‐injection using the designed US apparatus. (F) The protocol of the experimental timeline and the childhood Huh6 tumor treatment schedule. The mice in the control groups were intravenously injected with Abr (Paclitaxel: 10 mg kg^−1^), Oni (Irinotecan: 7.5 mg kg^−1^), and Doxil (Doxorubicin: 4 mg kg^−1^) and then with US irradiation at 6 h post‐injection (n = 5). The changes in (G) the tumor volumes and (H) body weight during the treatments, and (I) the photographs of representative mice from each group at the end of the treatment. (J) Photograph of the excised tumors and average tumor weight of each group at the end of the Huh6 tumor treatment experiment. (K) Histological analysis of tumors after H&E staining, and IHC staining of Ki67 (scale bar = 100 µm). (L) Quantitative analysis of the integrated optical density of fluorescence in the Ki67 IHC staining images (n = 10). Ten pictures in each group were randomly selected and analyzed using Image‐J software. Data were expressed as the mean ± SD, significances were determined by one‐way ANOVA with Tukey's correction, *ns* means no significant difference, ^∗^ *p* < 0.05, ^∗∗^ *p* < 0.01, ^∗∗∗^ *p* < 0.001.

As the water‐insoluble CPT was hardly used through intravenous injection, the blood clearance of CSCPTL was investigated in childhood tumor‐bearing mice by comparing it with a clinically widely used CPT derivative of 10‐hydroxycamptothecine (HyCPT), ensuring comparability in terms of potential clinical translation. The blood samples were collected via the orbital venous plexus at timed intervals within a 12‐h period, and the drug concentration in the blood was extracted and measured by HPLC (Figure 4A). The drug concentration of HyCPT in the bloodstream sharply decreased and was hardly detectable beyond 6 h, whereas that of CSCPTL gradually reduced and could be detected even at 12 h. According to the calculated pharmacokinetic parameters of CSCPTL, its elimination half‐time (*T_1/2_*) was approximately 1.88 ± 0.23 h, the area under the cumulative curve (AUC) was approximately 1.35 ±0.17 mg mL^−1^ h^−1^, and the mean residence time (MRT) was as high as 5.90 ± 0.83 h, which was significantly higher than that of HyCPT (0.52 ± 0.15 h for *T_1/2_*, 0.23 ± 0.07 mg mL^−1^ h^−1^ for AUC, and 0.66 ± 0.14 h for MRT), thereby providing sufficient circulated drug amount and time for tumor accumulation of CSCPTL. The in vivo and *ex vivo* biodistribution of CSCPTL was measured by using the IVIS Spectrum System at 24 h after injection (Figure 4B). Although the fluorescence accumulation of CSCPTL in the tumor was considerable, the liver and spleen exhibited higher fluorescence accumulation compared to other tissues and tumors, which corresponded with the off‐target distribution of the liver and spleen capture. The drug concentration of the tumor in the CSCPTL group was approximately 3.4‐fold that of the tumor in the HyCPT group, and CSCPTL could reduce the off‐target distribution of the lung, kidney, and intestine in comparison with HyCPT, but the drug concentration of the liver and spleen in the CSCPTL group was significantly higher than that of the liver and spleen in the HyCPT group, indicating the limitations of nanocarriers.

We then tested the in vivo antitumor activities of the liposomes on childhood HepG2 tumor‐bearing mice. The mice with a tumor volume of approximately 50 mm^3^ were selected, grouped, and intravenously administered with HyCPT, SCPTL, and CSCPTL (equivalent to CPT dose 5 mg kg^−1^) or control of CL and PBS for a total of three times (Figure 4C), and then subjected to US irradiation (intensity: 1 W cm^−2^, frequency: 3 MHz, duty cycle: 50%, duration: 10 min, Mettler Sonicator‐740) at 6 h after injection using the designed US apparatus (Figure 4D). At the end of the experiment, the mice were euthanized, and the tumors were collected, photographed, and weighed (Figure 4E). CSCPTL exhibited an average tumor inhibition rate of 88.9%, which was much higher than those of CL (23.6%), HyCPT (62.2%), and SCPTL (60.7%), initially proving the better anticancer activities of CSCPTL on childhood solid tumors.

In order to explore the clinical translational potential of CSCPTL on childhood solid tumor treatment, an extensive investigation was conducted to test the anticancer activities, biocompatibility, and safety by comparing with three widely‐used clinical nanodrugs, Abraxane (Abr, a form of paclitaxel protein‐bound), Onivyde (Oni, a liposomal formulation of irinotecan), and Doxil (PEGylated liposomal doxorubicin) on another childhood Huh6 hepatoblastoma‐bearing mice [40, 41]. The doses of these nanodrugs were selected based on their clinical doses and the practice guide of dose conversion between animals and humans in terms of body weight. These mice were intravenously administered with HyCPT, CSCPTL (equivalent to CPT dose 5 mg kg^−1^), Abr (Paclitaxel: 10 mg kg^−1^), Oni (Irinotecan: 7.5 mg kg^−1^), Doxil (Doxorubicin: 4 mg kg^−1^), and PBS and were treated with US irradiation at 6 h post‐injection for a total of three times (Figure 4F and D). The mice treated with PBS or HyCPT exhibited evident continuous tumor growth during the treatment, whereas the tumor growth of the Abr, Oni, and Doxil groups was delayed during the treatment, but recovered to grow after the treatment. In contrast, the tumor growth of the CSCPTL group was substantially inhibited and continued to regress after the treatment (Figure 4G–I). The mice treated with Abr and CSCPTL displayed no noticeable side effects in terms of body weight change, whereas those of HyCPT, Doxil, and Oni exhibited body weight loss. All experimental mice were sacrificed, and their tumors were collected, photographed, and weighed. Some of the CSCPTL‐treated mice were cured and tumor‐free at the end of the experiment (Figure 4J). When compared to the PBS group, the Abr, Oni, Doxil, and CSCPTL groups exhibited respectable tumor inhibition. The tumor inhibition rate of CSCPTL was 95.3%, which was much higher than those of HyCPT (39.3%), Abr (58.1%), Oni (83.7%), and Doxil (67.6%), demonstrating the efficient therapeutic outcomes by utilizing the US‐responsive prodrug activation of CSCPTL liposome.

The antitumor mechanism was investigated by histological analyses and staining of the treated tumors (Figure 4K; Figure S15). Hematoxylin and eosin (H&E) staining revealed that the tumors treated with Abr, Oni, Doxil, and CSCPTL had much less densely populated cells than those treated with PBS and HyCPT. Moreover, CSCPTL‐treated tumors presented many more apoptotic cells with abundant nuclear shrinkage and extensive intercellular cavums owing to the ablation of tumor cells. Notably, when compared to the other groups, CSCPTL could significantly reduce Ki67‐positive tumor cells (brown signal) in the immunohistochemical staining (Figure 4L) and induce more numbers of apoptotic cells (green signal) in the TUNEL assay (Figures S15 A,B). Western blot analysis showed CSCPTL‐treated tumors could express more apoptosis‐related protein of activated Caspase‐3 to efficiently induce tumor cell apoptosis than those of HyCPT, Abr, Oni, and Doxil groups (Figure S15 C,D). These results collectively indicated a better prognosis after CSCPTL treatment.

**FIGURE 5:**
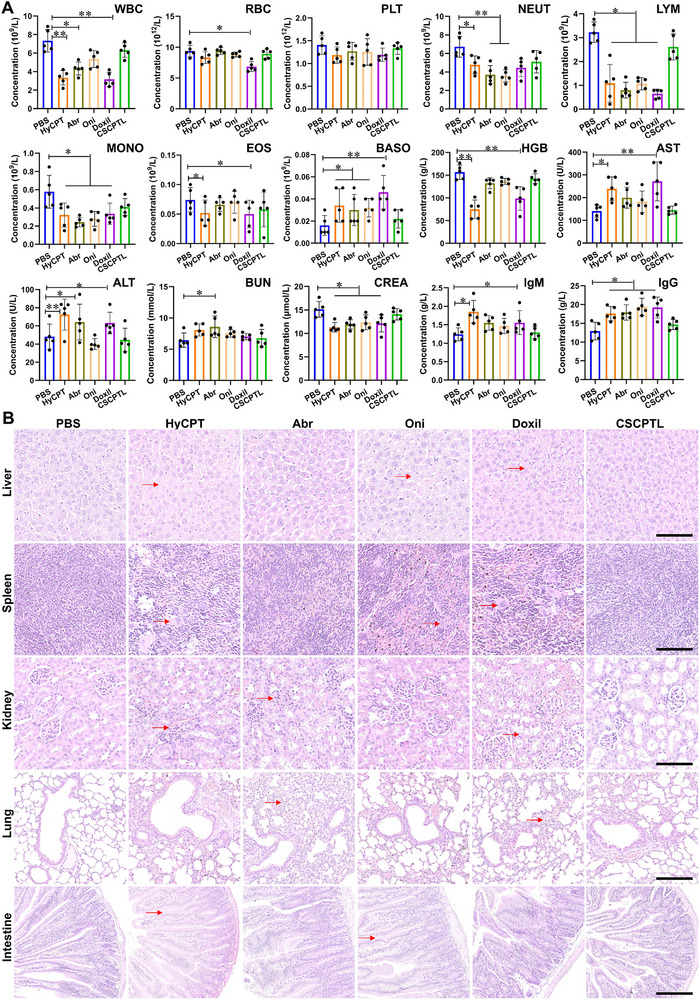
In vivo biocompatibility and biosafety evaluation. (A) The blood routine examination, serum biochemistry analysis, and immunogenicity assay were performed at the end of the experiments (WBC: white blood cells; RBC: red blood cells; PLT: blood platelets; NEUT: neutrophils; LYM: lymphocytes; MONO: monocytes; EOS: eosinophils; BASO: basophils; HGB: hemoglobin; AST: aspartate aminotransferase; ALT: alanine transaminase; BUN: blood urea nitrogen; CREA: serum creatinine; lgM: immunoglobulin M; lgG: immunoglobulin G). (B) Representative images of the H&E staining of the main organs (i.e., the liver, spleen, lung, kidney, and intestine). Some typical pathological changes (e.g., cellular necrosis, tissue vacuoles, inflammatory infiltration) are indexed with red arrows (scale bar = 100 µm). Data were expressed as the mean ± SD (n = 5), and significances were determined by one‐way ANOVA with Tukey's correction ^∗^ *p* < 0.05, ^∗∗^ *p* < 0.01.

To evaluate the biocompatibility and biosafety of liposomes, the blood routine examination, serum biochemistry analysis, and immunogenicity assay were conducted at the end of in vivo antitumor experiments [42, 43]. When compared with the PBS group, the juvenile mice treated with CSCPTL exhibited no significant changes in terms of hematological indexes of white blood cells (WBC), red blood cells (RBC), neutrophils (NEUT), lymphocytes (LYM), monocytes (MONO), eosinophils (EOS), basophils (BASO), hemoglobin (HGB), and serum biochemistry indexes of aspartate aminotransferase (AST), alanine aminotransferase (ALT), blood urea nitrogen (BUN), and creatinine (CREA), as well as immunogenicity indexes of immunoglobulin M (lgM) and immunoglobulin G (lgG) (Figure 5A). Even if some of the index values in the CSCPTL group were slightly increasing or decreasing while remaining within the acceptable healthy ranges with reference to the standard hematological and biochemical parameters data for BALB/c nude mouse colonies. However, the mice treated with HyCPT, Abr, Oni, and Doxil all displayed different levels of abnormal indices. Specifically, HyCPT and Doxil induced a significant decrease in terms of WBC, LYM, MONO, EOS, and HGB and a significant increase in terms of BASO, AST, ALT, lgG, and lgM, suggesting a high risk of safety, myelosuppression, hepatic toxicity, and immunological reaction for child patient treatment; Abr induced a significant change in terms of WBC, NEUT, LYM, MONO, BASO, ALT, BUN, and CREA, indicating an inflammatory response, myelosuppression, and renal functional damage; Oni sharply changed the hematological indexes of NEUT, LYM, MONO, and BASO, suggesting damage to the innate immune system and a higher risk of infection. Furthermore, the dissected organs were investigated using histological analyses of H&E staining (Figure 5B). The pathological images exhibited different extents of tissue damage in the HyCPT, Abr, Oni, and Doxil groups, as indexed with red arrows in the images. Obviously, the liver in HyCPT, Oni and Doxil groups exhibited extensive drug‐induced liver injury characterized by inflammation or necrosis of hepatocytes within the lobules; the spleen in HyCPT, Oni and Doxil groups displayed quite a few infarctions and necrosis of lymphoid follicle; the kidney in the HyCPT, Abr and Doxil groups showed enlarged glomerular volume, and severe granular and vacuolar degeneration of renal tubular epithelial cells; the lung in the Abr and Doxil groups displayed abnormal fibrosis and inflammation of the alveolar walls and pulmonary interstitium; the intestine in the HyCPT and Oni groups displayed ulcerative colitis, as characterized by epithelial cell abnormality, crypt abscesses, and mucosal inflammation. On the contrary, H&E staining images of the tissues in the CSCPTL group exhibited no significant pathological variations or adverse damages relative to those in the PBS group. These results collectively proved the good biosafety and biocompatibility of CSCPTL, ensuring its potential for efficient childhood solid tumor treatment with a prognosis of preventable adverse events.

While this US‐activated nanomedicine is promising for overcoming the age‐associated biodistribution disparity in pediatric solid tumor therapy, the clinical translation and commercialization of this strategy still face a lot of serious challenges. In terms of therapeutic efficiency, as there are significant inter‐ and intra‐tumor heterogeneities across different pediatric patients and tumor types and locations, the US‐activated nanomedicines should be precisely controlled under the real‐time contrast‐enhanced ultrasonography. On the other hand, in terms of safety and pediatric‐specific indication, as there are fundamental physiological differences between children and adults, the recruitment of representative clinical models of children for these comorbidities in children and adults should be legally approved by governmental drug administration, rather than adjusting the adult drug doses and US irradiation doses based on body weight or surface area to treat pediatric tumors.

## 3 Conclusions

In summary, we identified a clear age‐associated disparity in nanocarrier biodistribution characterized by significantly greater off‐target accumulation in pediatric solid tumors. We unveiled the underlying causes as the vigorous proliferation of neovascularization and heightened phagocytic activity of macrophages in juvenile models. To address this challenge, we proposed a novel concept: an exogenous stimuli‐responsive nano‐prodrug system that operates independently of endogenous tumor microenvironmental factors to overcome age‐related disparities of off‐target distribution. We demonstrated the feasibility and effectiveness of this approach through the development of an ultrasound‐activated liposomal prodrug (CSCPTL) for the treatment of pediatric solid tumors. Even when CSCPTL is unavoidably distributed to normal tissues, its pharmacologically inactive form exerts negligible effects on healthy cells. The liposomal prodrug can be activated through US irradiation in combination with a sonosensitizer, thus achieving robust antitumor efficacy across multiple juvenile hepatoblastoma mouse models and exhibiting excellent biocompatibility with no observable chemotherapy‐induced side effects. Overall, this study presents a compelling rationale for designing age‐appropriate cancer nanomedicines and establishes US‐activated liposomal prodrugs as a practical, effective, and side‐effect‐avoidable platform for pediatric solid tumor therapy.

## 4 Experimental Section

### 4.1 Animals

Male BALB/c nude mice (3–4‐week‐old for the child and 9–10‐week‐old for adult) were supplied by the Laboratory Animal Center of Zhejiang Chinese Medical University. Mice were housed in approved animal‐care facilities on a 12 h light/dark cycle and given ad libitum access to food and water. The mice were subcutaneously inoculated with 5 × 10^7^ HepG2 or Huh6 cells (0.1 mL cell suspension in the mixture of PBS and Matrigel Matrix) on their right flanks. Once the tumors reached approximately 50∼110 mm^3^ (7∼10 days after inoculation), mice were randomly assigned to different groups for animal experiments. The childhood solid tumor models with tumor volume 50∼60 mm^3^ and adulthood solid tumor models with tumor volume 90∼100 mm^3^ were selected to ensure the index ratio of tumor volume/body weight at the same level.

### 4.2 Age‐Associated Disparity of Nanocarrier Biodistribution in Childhood and Adulthood Solid Tumor Models

The in vivo fluorescent imaging and biodistribution of the three selected fluorescent‐labeled nanocarrier models (liposome, BSA, and micelle) were respectively performed in the 4‐week‐old and 10‐week‐old HepG2 tumor‐bearing mice. The mice were intravenously injected with the Cy5‐labeled carriers (Equivalent fluorescence intensity 5 000 units mL^−1^, 100 µL 10 g^−1^). Whole‐body optical imaging was performed at 24 h of post‐injection on a fluorescent spectral imager of Caliper IVIS Lumina II (PerkinElmer, USA) equipped with fluorescent filter sets (excitation/emission, 640/670 nm). The mice were sacrificed, tumors as well as major organs including heart, liver, spleen, lung, kidneys, intestine, and muscle were collected, weighed, and subjected to *ex vivo* imaging using the same parameters described above. The fluorescence intensity of tissue distribution was quantitatively analyzed by Living Image‐4.5 software in terms of absolute intensity of total units/tissue weight.

### 4.3 Western‐Blotting Analysis of Tissues

The tissues of childhood and adulthood tumor‐bearing mice were dissected separately and lysed using the protein extraction reagent RIPA (Invitrogen, Carlsbad, CA) supplemented with phenylmethanesulfonyl fluoride (PMSF, Roche). After grinding, the tissue lysate was centrifuged to obtain its supernatant. Equivalent amounts of proteins (50 µg) from each sample were electrophoresed on a 4–20% SDS‐polyacrylamide gel (SDS‐PAGE) and then transferred to a polyvinylidene fluoride membrane, blocked in 5% BSA solution for 2 h at room temperature, and incubated with the following specific primary antibodies: rabbit anti‐human VEGF Receptor 1 antibody (1:1000, #ab32152, Abcam), rabbit anti‐human CD31 antibody (1:1000, #ab222783, Abcam), and loading control of rabbit anti‐human β‐Actin antibody (1:2000, # ab8226, Abcam). HRP‐linked secondary antibody is goat anti‐rabbit IgG (1: 5000; CST, Boston, USA). Pre‐stained Color Protein Standard Marker (10–250 kDa, Thermo Fisher Scientific) was used as a molecular marker. A hypersensitivity ECL chemiluminescence kit (Beyotime Biotechnology, China) was used to detect specific bands, and the PVDF membrane was analyzed using the Azure c600 Imager (Azure Biosystem, USA).

### 4.4 Fabrication and Characterization of Liposomes

Taking the CSCPTL liposome for example, a 10 mg lipid mixture consisting of SCPT (2.0 mg, 1.46 µmol), Ce6‐PEG2000‐DSPE (4 mg, 1.16 µmol), RGD‐PEG2000‐DSPE (1.0 mg, 0.30 µmol), HSPC (2.0 mg, 2.63 µmol), and cholesterol (1.0 mg, 2.72 µmol) was added to a 10 mL flask and dissolved in chloroform (3 mL). The mixture was evaporated for 30 min using a rotary evaporator and vacuumed for 1 h using a vacuum drying chamber to form a lipid film at room temperature. The lipid films were rehydrated with 5 mL PBS and mechanically agitated for 1 h at 4°C. Similarly, with everything else being the same as the CSCPTL fabrication, two control liposomes, including the non‐drug loading (i.e., without SCPT component) liposome (CL) and the non‐sonosensitizer loading (i.e., without Ce6‐PEG2000‐DSPE component) liposome (SCPTL), were parallelly prepared (Table S1). At the same time, the Cy5‐labeled liposomes were prepared by adding Cy5‐labeled PEG2000‐DSPE (0.05 mg, 0.015 µmol, 0.5% mass ratio of the total lipids) into the prescription as the method mentioned above. The hydrodynamic diameter size and zeta potentials of particles were measured using a dynamic light scattering analyzer (Nano‐ZS 90, Malvern) following proper dilution with PBS or serum‐containing medium at 25°C. The morphology of nanosized particles was imaged by cryo‐TEM (Talos F200C 200kv, FEI Inc.) in a carbon‐coated 200‐mesh copper TEM grid. The fluorescence spectra and fluorescence intensity of Cy5‐labeled nanodroplets were detected with a microplate reader (SpectraMax M4, Molecular Devices). The liposome suspension (1 mL) was dialyzed (dialysis tubing type: MEMBRA‐CEL MD44, Mw cut‐off 3.5 kDa) in 100 mL PBS solution (containing 5 vol% glycerol) for 12 h, and the concentration of CPT in the dialysate was determined by HPLC.

### 4.5 Intracellular ROS Assessments

Intracellular ROS production was examined by 2′,7′‐dichlorodihydrofluorescein diacetate (DCFH‐DA; 10 µM) as a probe using a reactive oxygen species assay kit. For flow cytometry analysis, HepG2 cells were seeded into 12‐well plates and incubated overnight. Following 6 h incubation with liposomes, cells were washed in PBS and loaded with 10 µM oxidant‐sensitive dye DCFH‐DA in serum‐free medium for 20 min. Subsequently, the cells were performed with US irradiation (US parameter: 3 MHz, 50% of duty cycle for 5 min) and immediately washed twice. Cells were resuspended and transferred into the tube. The fluorescence intensity of intracellular DCFH‐DA was quantified by a Beckman CytoFlex flow cytometer. The DCFH‐DA fluorescence images were acquired through a confocal laser scanning microscope (CLSM). HepG2 cells were seeded on 35 mm glass‐bottom dishes, incubated for 24 h, and then processed as described above until the completion of DCFH‐DA staining. After washing 3 times with cold PBS, intracellular ROS production images were obtained with a CLSM at an excitation wavelength of 488 nm and emission wavelength of 523 nm channel.

### 4.6 Cellular Uptake and Subcellular Distribution

HepG2 cells were seeded in 12‐well plates and cultured for 24 h. Liposomes with equal fluorescence intensity was added and incubated at 37°C for 0, 0.5, 1, 2, 3, 6 h. The cells were washed and harvested using 0.25% Trypsin‐EDTA and further centrifuged at 1000 rpm for 5 min. After washing and resuspended, the intracellular fluorescence of each sample was detected by flow cytometry. Each sample collected 10 000 gated events, and the data were analyzed using CellQuest Pro software. HepG2 cells were cultured on 35 mm glass‐bottom dishes for 48 h. The medium was replaced with 1 mL fresh serum‐free medium containing CSCPTL and incubated at 37°C for 0.5, 1, 2, and 4 h. Subsequently, cells were washed with PBS. The cells were further stained with LysoTracker Green (0.2 µL per dish, 30 min) and Hoechest 33342 (2 drops per dish, 15 min) to label lysosomes and nuclei, and stained with DIO (1 µL per dish, 30 min) and Hoechest 33342 (2 drops per dish, 15 min) to visualize the spatial location of cytomembrane, nuclei, and liposomes. The subcellular distribution images of liposomes were acquired with CLSM using 405, 488, and 640 nm wavelength channels, separately.

### 4.7 Cytotoxicity Test

The cytotoxicity of prodrugs and liposomes was evaluated by the CCK8 kit on the HepG2 and Huh6 cell lines. Briefly, cells were seeded in 96‐well plates at a density of 5000 cells per well, and 96 wells of each plate were subdivided into 10 groups (longitudinal six wells as a group) in terms of the application of the ultrasonic transducer. After incubated overnight, cells were exposed to different processes: incubation with various concentrations (nine serial dilutions of equivalent CPT in six replicates from 0 to 10 µm) of free drug and liposomes without US irradiation, or treatment with US irradiation (1 W cm^−2^, 3 MHz, 50% duty cycle, 5 min) after 6 h incubation, and then the samples were incubated for an additional 48 h. According to the CCK8 cell proliferation assay protocol, 10 µL of CCK8 solution and 90 µL DMEM were added to each well. After 1 h, optical density was measured at 450 nm using a microplate spectrophotometer. Cell viability after different treatments was calculated as the percentage of the absorbance in untreated cells. In addition, the cytotoxicity of prodrugs and liposomes were also evaluated on two normal cell lines of NIH/3T3 (a fibroblast cell line) and HK2 (a proximal tubular cell line) as described above.

### 4.8 Apoptosis Test

Apoptosis and cell death were assayed via FITC Annexin‐V and PI double staining using Annexin V‐FITC Apoptosis Detection Kit following the manufacturer's protocol. HepG2 cells were seeded into 12‐well plates and incubated overnight for subjected to various liposomes with or without US treatment, and further incubation for 48 h. CPT‐equivalent concentration is 0.5 µM. After washed with PBS and collected by trypsin digestion, the cells were counted and stained with Annexin V‐FITC and PI at room temperature in the dark, followed by flow cytometry analysis.

### 4.9 Biodistribution and In Vivo/Ex Vivo Imaging

Childhood HepG2 tumor‐bearing mice were used to evaluate the biodistribution and tumor accumulation of HyCPT and CSCPTL (dose equivalent to CPT 5 mg kg^−1^, 3 mice in each group). When tumors reached about 80 mm^3^, the mice were intravenously injected with HyCPT and CSCPTL. The fluorescent signal was observed at an excitation of 640 nm and emission of 670 nm at 24 h after injection. The mice were sacrificed, tumors as well as major organs, including heart, liver, spleen, lung, kidneys, and intestine, were collected, weighed, and subjected for *ex vivo* imaging using the same parameters described above.

The fluorescence intensity and drug concentration of tissue accumulation were quantitatively analyzed using Living Image‐4.5 software and HPLC.

### 4.10 In Vivo Antitumor Efficacy

In the initial in vivo antitumor experiment, the childhood HepG2 tumor‐bearing mice were intravenously injected with HyCPT, SCPTL, and CSCPTL (equivalent to CPT dose 5 mg kg^−1^), or blank control CL and PBS, and treated with US irradiation (intensity: 1 W cm^−2^, frequency: 3 MHz, duty cycle: 50%, duration: 10 min) at 6 h post‐injection using the designed US apparatus. The treatment was performed by intravenous injection every 2 days for a total of 3 times. Body weight, tumor size, and animal condition were monitored at regular intervals. Mice were sacrificed at 12 days after the first treatment. Resected tumors and major organs were weighted and fixed in 4% paraformaldehyde fix solution for 24 h before embedding in paraffin. The inhibition rate of tumor growth (IRT) was calculated as follows: IRT = 100% × (mean tumor weight of the control group – mean tumor weight of the experimental group) / mean tumor weight of the control group. The therapeutic efficacy of the treatments was evaluated by comparing the experimental group with the PBS group.

In order to explore the clinical translational potential of CSCPTL on childhood solid tumors, an extensive investigation was further conducted to test the anticancer activities, biocompatibility, and safety by comparing with three widely‐used clinical nanodrugs, Abraxane (Abr, a form of paclitaxel protein‐bound), Onivyde (Oni, a liposomal formulation of irinotecan), and Doxil (a PEGylated liposomal doxorubicin) on another childhood Huh6 hepatoblastoma‐bearing mice. The doses of Abr, Oni, and Doxil were selected based on the clinical doses and practice guide of dose conversion between animals and humans in terms of body weight. The mice were intravenously administered with HyCPT, and CSCPTL (equivalent to CPT dose 5 mg kg^−1^), and Abr (Paclitaxel: 10 mg kg^−1^), Oni (Irinotecan: 7.5 mg kg^−1^), Doxil (Doxorubicin: 4 mg kg^−1^) and PBS, and treated with US irradiation (intensity: 1 W cm^−2^, frequency: 3 MHz, duty cycle: 50%, duration: 10 min) at 6 h post‐injection for total three times. Body weight, tumor size, and animal condition were monitored at regular intervals. At the end of the experiment on day 12, whole blood was sampled and anticoagulated with EDTA for the blood routine assay to evaluate the chemotherapy‐induced adverse effects using an automated hematology analyzer (Sysmex, USA). At the same time, a portion of the blood was collected without EDTA in EP tubes. After 30 min resting, the supernatant serum was separated via 3000 rpm centrifuged for 10 min at 4°C for biochemistry testing using a full‐automatic biochemical analyzer (HITACHI, Japan). Then mice were sacrificed, and the resected tumors and major tissues were fixed in 4% paraformaldehyde for histopathological examinations and lysed for western blotting. The inhibition rate of tumor growth (IRT) was calculated. The therapeutic efficacy of the treatments was evaluated by comparing the experimental group with the PBS group.

### 4.11 Statistical Analysis

Unless otherwise stated, all data were presented as mean ± standard deviation (SD) from n ≥ 3 independent biological replicates and mice. The selection of microscopic images and the grouping of antitumor experiments are agreed with the principle of randomness. The images were quantitatively analyzed using Image J software. Graphing and statistical analyses were performed using GraphPad Prism 9. Statistical significance was determined with an unpaired two‐tailed Student's *t*‐test for continuous variables or one‐way ANOVA with Tukey's correction and 95% confidence intervals for categorical variables. For all tests, ns meant not significant, and the statistical tests with the value of ^*^ *p* < 0.05, ^**^ *p* < 0.01, and ^***^ *p* < 0.001 were considered statistically significant.

## Author Contributions

D.C., J.X., M.Y., J.H., and X.X. planned the experiments, designed and prepared figures, and performed the majority of the experiments. D.C., J.C., and H.C. wrote and edited the manuscript. Z.Z. and G.W. supervised the research and reviewed the manuscript. D.C. and J.X. contributed equally to this work. All authors reviewed the final version and approved the submission.

## Conflicts of Interest

The authors declare no conflict of interest.

## Ethics Statement

All experiments involving animals (License No. IACUC‐20241028‐12) were approved by the Institutional Animal Ethics Committee of the Laboratory Animal Center of Zhejiang Chinese Medical University.

## Supporting information




**Supporting File**: advs73530‐sup‐0001‐SuppMat.docx.

## Data Availability

The data that support the findings of this study are available from the corresponding authors upon reasonable request.
