# Supplementary material for: Ultrasound‐Boosted Liposomal Prodrug Overcomes Age‐Associated Biodistribution Disparity in Pediatric Solid Tumor Therapy
Source: Adv Sci (Weinh). 2025 Dec 27;13(14):e13148. doi: 10.1002/advs.202513148 (PMC12970182; doi:10.1002/advs.202513148)
Supplement: Supplementary file 1 — Supporting File: advs73530‐sup‐0001‐SuppMat.docx. [file ADVS-13-e13148-s001.docx]

***Supporting Information***

**Ultrasound-Boosted Liposomal Prodrug Overcomes Age-Associated Biodistribution Disparity in Pediatric Solid Tumor Therapy**

Danfei Chen, Junjun Xu, Jian Chen, Jue Hu, Xiaobo Xuan, Haifang Cai, Mingdong Yang,^*^ Zhuxian Zhou,^*^ and Guowei Wang^*^

Dr. D. Chen, Prof. J. Chen, J. Hu, X. Xuan, H. Cai

Department of Pediatrics, The First Affiliated Hospital of Zhejiang Chinese Medical University (Zhejiang Provincial Hospital of Chinese Medicine), Zhejiang Chinese Medical University, Hangzhou 310006, China.

J. Xu, Dr. M. Yang

Department of Pharmacy, The Second Affiliated Hospital of Zhejiang University School of Medicine, Zhejiang University, Hangzhou 310009, China.

E-mail: mingdongyang@zju.edu.cn

Prof. Z. Zhou, Prof. G. Wang

Zhejiang Key Laboratory of Smart Biomaterials and Key Laboratory of Biomass Chemical Engineering of Ministry of Education, College of Chemical and Biological Engineering, Zhejiang University, Hangzhou 310027, China.

E-mail: zhouzx@zju.edu.cn, wangguowei@zju.edu.cn

Prof. Z. Zhou, Prof. G. Wang

Department of Ultrasound in Medicine, The Second Affiliated Hospital of Zhejiang University School of Medicine, Zhejiang University, Hangzhou 310009, China.

E-mail: zhouzx@zju.edu.cn, wangguowei@zju.edu.cn

**Materials**

All chemical reagents other than indicated were purchased from Sigma-Aldrich Inc. or Aladdin Reagent Inc. Hydrogenated soybean phosphatidylcholine (HSPC), cholesterol, 1,2-distearyl-sn-glycerol-3-phosphoethanolamine-polyethylene glycol-2000 (PEG2000-DSPE), 1,2-Dioleoyl-sn-glycero-3-phosphoethanolamine (DOPE), Chlorin e6 (Ce6)-conjugated PEG2000-DSPE (Ce6-PEG2000-DSPE), Cyanine5 (Cy5)-labeled PEG2000-DSPE (Cy5-PEG2000-DSPE), and RGD peptide-conjugated PEG2000-DSPE (Cy5-PEG2000-DSPE) were purchased from Xi’an Ruixi Biological Technology Co., Ltd. DMEM medium, fetal bovine serum (FBS) and 0.25% trypsin solution were purchased from GIBCO. The Cell Counting Kit-8 (CCK-8) proliferation assay kit and Reactive Oxygen Species Assay Kit were purchased from Beyotime Biotechnology. Hoechst 33342, LysoTracker® Green DND26 and 3,3'-dioctadecyloxacarbocyanine perchlorate were purchased from Thermo Fisher Scientific Inc. TUNEL Apoptosis Assay Kit was purchased from Roche, BD Pharmingen. Ki67 antibody was purchased from Proteintech Group. Annexin V-FITC/PI Apoptosis Detection Kit was purchased from Solarbio Science & Technology Co., Ltd. The protein detection, urea nitrogen detection, and creatinine detection ELISA kits were purchased from Nanjing Jiancheng Bioengineering Institute (Nanjing, China). The primary antibodies of CD31 (#ab222783) and VEGFR1 (#ab32152) were purchased from Abcam company. The liposome model was fabricated using the lipid mixture of HSPC, cholesterol, PEG2000-DSPE and Cy5- PEG2000-DSPE in a molar ratio of 56.5 : 38.2 : 5.3 : 0.1, as described as the published Doxil formula.^[1]^ The BSA nanoparticle model was fabricated using the Cy5-conjugated BSA, and the micelle model with was fabricated using Cy5-conjugated poly (ethylene glycol 2000)-polystyrene 2000 block polymer based on our previous work.^[2]^

**Cell culture**

The human hepatoblastoma cell lines of Huh6 (RRID: CVCL_4381) and HepG2 (RRID: CVCL_0027) were purchased from American Type Culture Collection (ATCC, Manassas, VA, USA), and preserved in the National Collection of Authenticated Cell Cultures (Shanghai, China). Both of the cell lines were authenticated by Short Tandem Repeat (STR) profiling and confirmed to be contamination-free. Cells were maintained in DMEM medium supplemented with 10% FBS, penicillin (100 units mL^-1^) and streptomycin (100 μg mL^-1^) in a humidified atmosphere of 5% CO_2_ at 37 °C.

**Synthesis of CPT-DOPE conjugate containing thioketal linker (****SCPT) and CPT-DOPE conjugate containing uncleavable hexanediol linker (HCPT)**

The synthesis route is shown in **Figure S4**, based on our previous work.^[3]^ Camptothecin (CPT, 2.0 g, 5.74 mmol) and DMAP (2.11 g, 17.3 mmol) were suspended in dry DCM (50 mL) under argon atmosphere. Triphosgene (0.567 g, 1.92 mmol) was added and the mixture was stirred for 30 min at room temperature. Thioketal acrylate (1.58 g, 6.31 mmol, in 15 mL dry THF) was added dropwise *via* a constant pressure funnel. The reaction mixture was stirred overnight during which a white precipitate was formed. After filtration and evaporating all the solvents, the residues were diluted with diethyl acetate and washed once with water, twice with 1.0 M HCl, and twice with brine, respectively. The organic layer was dried over anhydrous MgSO4, filtered, and concentrated on a rotary evaporator. The crude product was purified by column chromatography using ethyl acetate as eluent (100% EtOAc, visualize under UV light) to give CPT-SS-acrylate as a pale solid powder (2.92 g, yield: 86%). CPT-CC-acrylate was synthesized by using the same method above-mentioned. CPT-SS-acrylate: ^1^H NMR (400 MHz, CDCl_3_, *δ*): 8.42 (s, 1H), 8.26 (s, 1H), 7.96 (s, 1H), 7.85 (s, 1H), 7.69 (s, 1H), 6.39 (s, 1H), 6.08 (s, 1H), 5.84 (s, 1H), 5.73 (s, 1H), 5.38 (d, J = 35.3 Hz, 3H), 4.25 (s, 4H), 2.85 (s, 3H), 2.23 (d, J = 32.9 Hz, 2H), 1.55 (s, 5H), 1.02 (s, 3H). CPT-CC-acrylate: ^1^H NMR (400 MHz, CDCl_3_, *δ*): 8.42 (s, 1H), 8.25 (s, 1H), 7.94 (s, 1H), 7.87 (s, 1H), 7.70 (s, 1H), 7.36 (s, 1H), 6.34 (s, 1H), 6.09 (s, 1H), 5.73 (d, *J* = 40.4 Hz, 2H), 5.42 (s, 1H), 5.30 (s, 2H), 4.11 (s, 4H), 2.21 (d, *J* = 61.7 Hz, 2H), 1.62 (s, 6H), 1.38 (s, 4H), 1.02 (s, 3H). CPT-SS-acrylate and CPT-CC-acrylate could be authenticated by the molecular weight of [M+H]^+^ peaks in MALDI-TOF-MS spectra. CPT-SS-acrylate and CPT-CC-acrylate were 625.1 and 547.2 respectively.

CPT-SS-acrylate (63 mg, 100 µmol) and DOPE (75 mg, 100 µmol) were dissolved in DMF (2 mL) and stirred at 45 ^o^C. The reaction was monitored by the HPLC. Above 90% of CPT-SS-acrylate was conjugated with DOPE at 24 h, then the solution was purified by dialysis (Mw cut-off 1.0 kDa) in methanol (volume ratio of sample to dialysis buffer was 1/100) for 24 h by replacing the fresh methanol every 6 h. After drying in rotary evaporator, the SCPT was obtained (*ca.* 90 mg, 65.3% yield) as characterized by ^1^H-NMR and MALDI-TOF-MS spectra (**Figure S5 and S6**). Parallelly, HCPT was synthesized by conjugating CPT-CC-acrylate wiht DOPE using the same method (**Figure S7 and S8**).

**ROS-responsive prodrug activation**

SCPT or HCPT (10 μM) were respectively dissolved and incubated in the mixture solution of DMSO/H_2_O (1/1 v/v) containing 1 mM H_2_O_2_ at 37 °C. At timed intervals, 100 μL of the solution was sampled and subjected to high performance liquid chromatography (HPLC). HPLC consisted of a 1525 binary pump, 2475 multi-*λ*-fluorescence detector and 2998 photodiode array detector and a SunFireTM C_18_ (4.6 × 250 mm, 5 μm) column. The mobile phase was using methanol / water = 60 / 40 (v/v) at a flow rate of 1.0 mL/min at 35 °C.

**In vitro stability of liposomes**

Liposomes were diluted with PBS buffer or RPMI 1640 medium. The mixture was incubated at 25°C for different times. After incubation for a timed interval, a 100 μL solution was sampled and diluted with 900 μL PBS buffer. The changes of particle size and SCTP content in different temperatures of 4°C, 25°C, and 37°C for 48 h in the dark condition were measured using the Zeta-Nanosizer and HPLC as described above. The changes of particle size and SCPT content in different light exposures of 0, 2 000 Lux, 4 000 Lux, and 6 000 Lux at 4°C for 48 h was also tested using the light stability test chamber equipped with both temperature and light adjuster (Labonce-150CGS-FC, Beijing Labonce Themostatic Technology Company, China).

**Blood clearance**

As the water-insoluble CPT was hardly be used through intravenous injection, the blood clearance of CSCPTL was investigated in childhood tumor-bearing mice by comparing with a clinical widely-used CPT derivative of 10-hydroxycamptothecine (HyCPT), ensuring comparability in terms of potential clinical translation. The childhood HepG2 tumor-bearing mice were intravenously injected with HyCPT and CSCPTL (dose equivalent to CPT 5 mg kg^-1^, 3 mice in each group). Blood samples (50 µL) were collected *via* the orbital venous plexus of mice at timed intervals (5 min, 0.5 h, 1 h, 2 h, 4 h, 8 h, 12 h, and 24 h) and mixed with heparin solution (1 mg mL^-1^, 50 µL). The samples were separated from the blood by centrifuge at 5,000 rpm for 5 min at 4 °C. The samples were then diluted with acetonitrile (900 µL). The mixtures were thoroughly vortexed, and centrifuged at 5, 000 rpm for 5 min. Next, 500 µL of supernatant was removed and concentrated for HPLC analysis. The drug content was calculated according to the standard curve established previously in our lab, and pharmacokinetic parameters were analyzed.

**Histological assay**

The tissues were fixed with 4% neutral buffered paraformaldehyde and embedded in paraffin. Tissue sections of 5-μm thick were mounted onto glass slides and stained with hematoxylin-eosin (H&E) and examined by light microscopy (OLYMPUS, BX51, Japan). Tissue sections were subjected to Ki67 staining using the Ki67-antibody Assay Kit (Proteintech, USA) according to the manufacturer’s protocol. Apoptotic events were also determined by the TdT-mediated dUTP nick end labeling (TUNEL) assay. Tissue sections were subjected to TUNEL staining using the TUNEL Apoptosis Assay Kit (Roche, Switzerland) according to the manufacturer’s protocol. Apoptotic cells were identified by positive TUNEL staining and were examined by CLSM. The apoptosis-related protein of activated caspase 3 (#25128-1-AP, Proteintech Group) was further analyzed using western blotting.

**Figures and Tables**


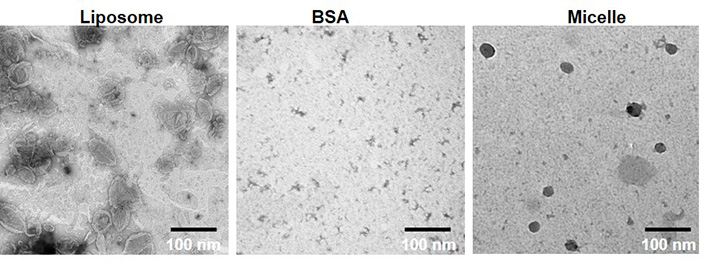


**Figure S1**. The transmission electron microscope images of the fluorescent-labeled nanocarrier models: liposome, bovine serum albumin (BSA), and micelle.


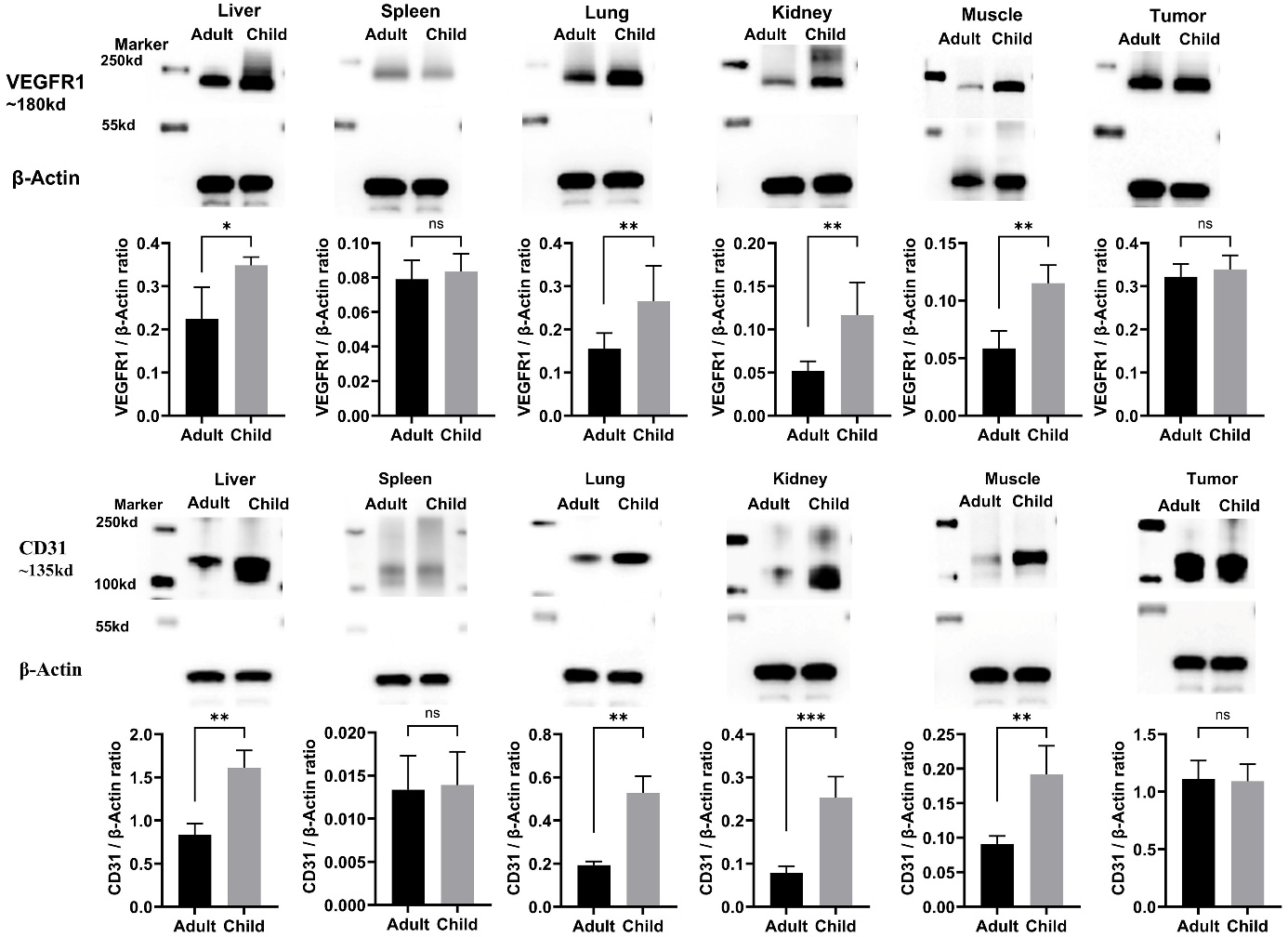


**Figure S2**. The western blotting analysis of neovascularization in childhood and adulthood solid tumor models in terms of the typical expressing biomarkers: vascular endothelial growth factor receptor-1 (VEGFR1) and platelet endothelial cell adhesion molecule-1 (CD31). The primary antibodies of VEGFR1 (#ab32152) and CD31 (#ab222783) are purchased from Abcam company and used according to the manufacturer's instructions. Data are expressed as mean ± SD (n = 3), significances are determined by unpaired t-test, ^*^: *P* < 0.05, ^**^: *P* < 0.01, and *ns* means no significant difference.


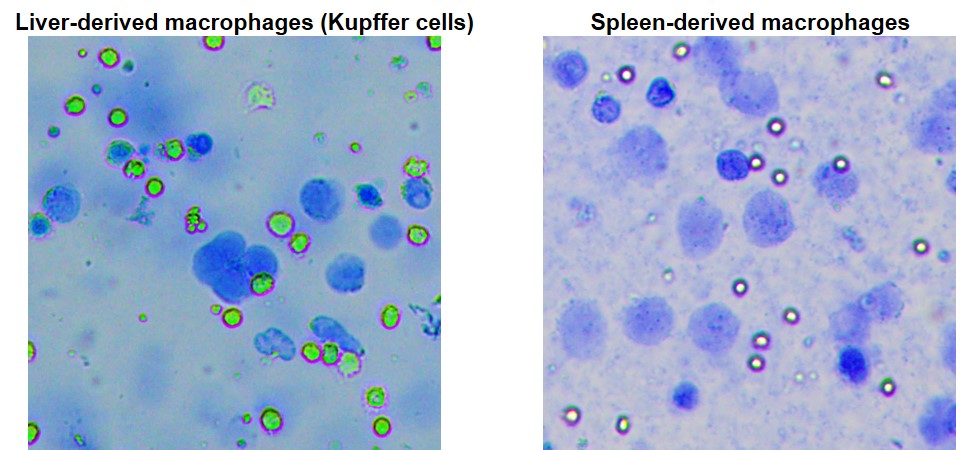


**Figure S3.** The macrophages were extracted from liver and spleen using adherent screening method and confirmed using Trypan Blue Staining. The living macrophages can phagocytose the dye of Trypan Blue and quickly adhere on the dish wall, and imaged using the light microscope.





**Figure S4.** Synthetic procedure of (A) the CPT prodrug containing ROS-cleavable thioketal linker (CPT-SS-acrylate) and CPT prodrug containing uncleavable hexanediol linker (CPT-CC-acrylate), and (B) CPT-DOPE conjugate containing thioketal linker (SCPT) and CPT-DOPE conjugate containing uncleavable hexanediol linker (HCPT)

**
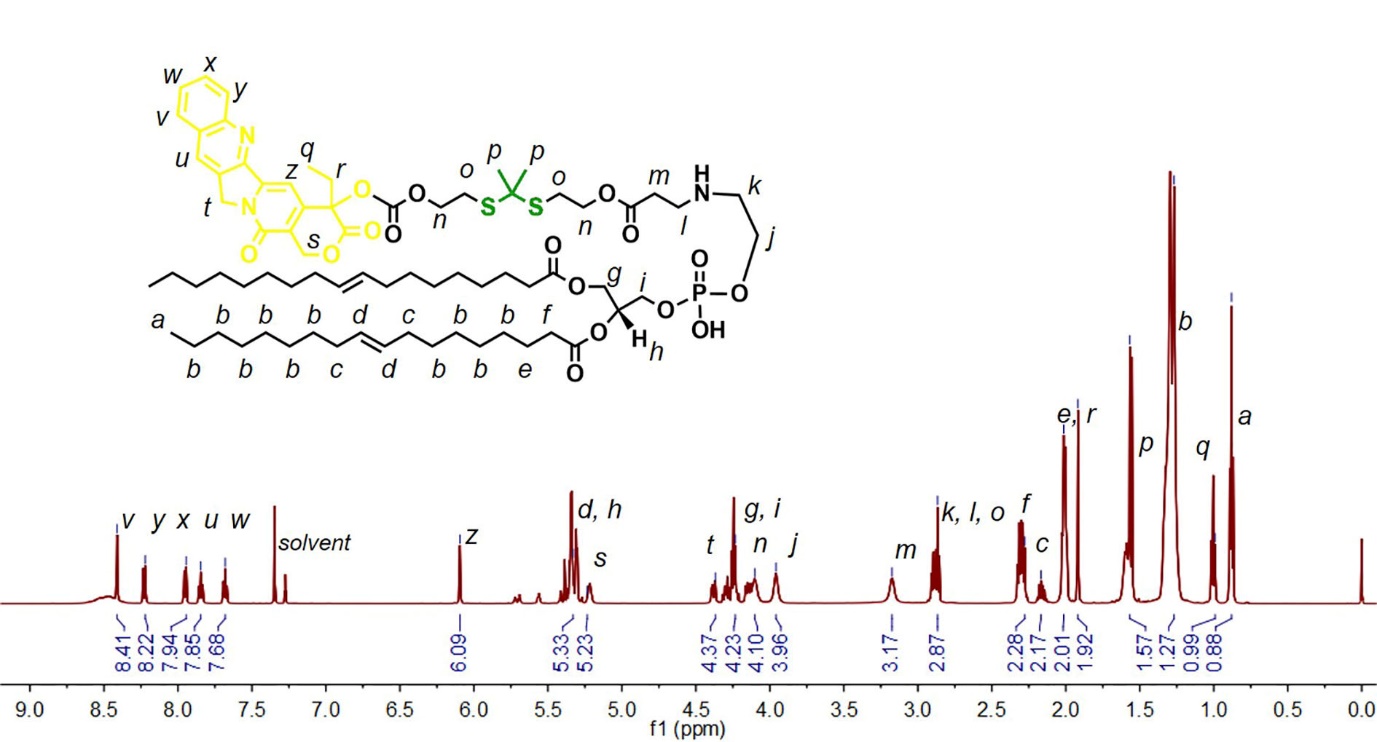
**

**Figure S5.** The ^1^H NMR spectrum of camptothecin-DOPE prodrug conjugate containing reactive oxygen species-cleavable thioketal linker (SCPT) in CDCl_3_ solvent.


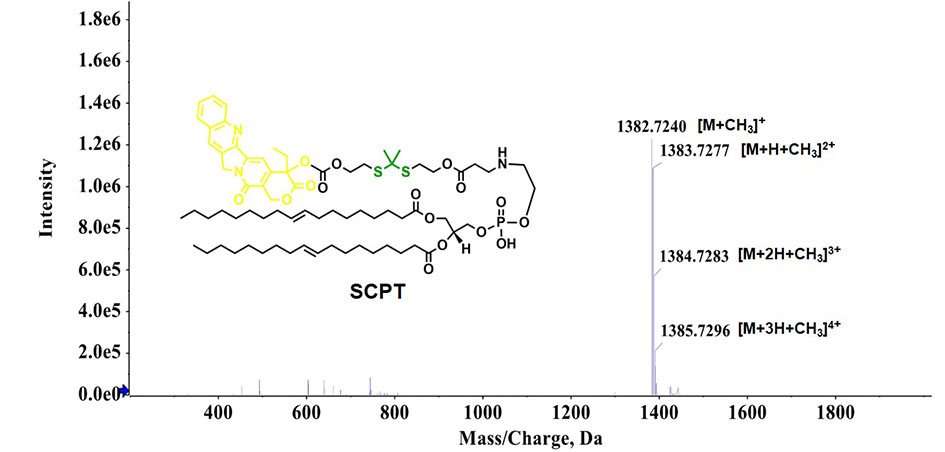


**Figure S6.** The MALDI-TOF-MS spectrum of SCPT.

**
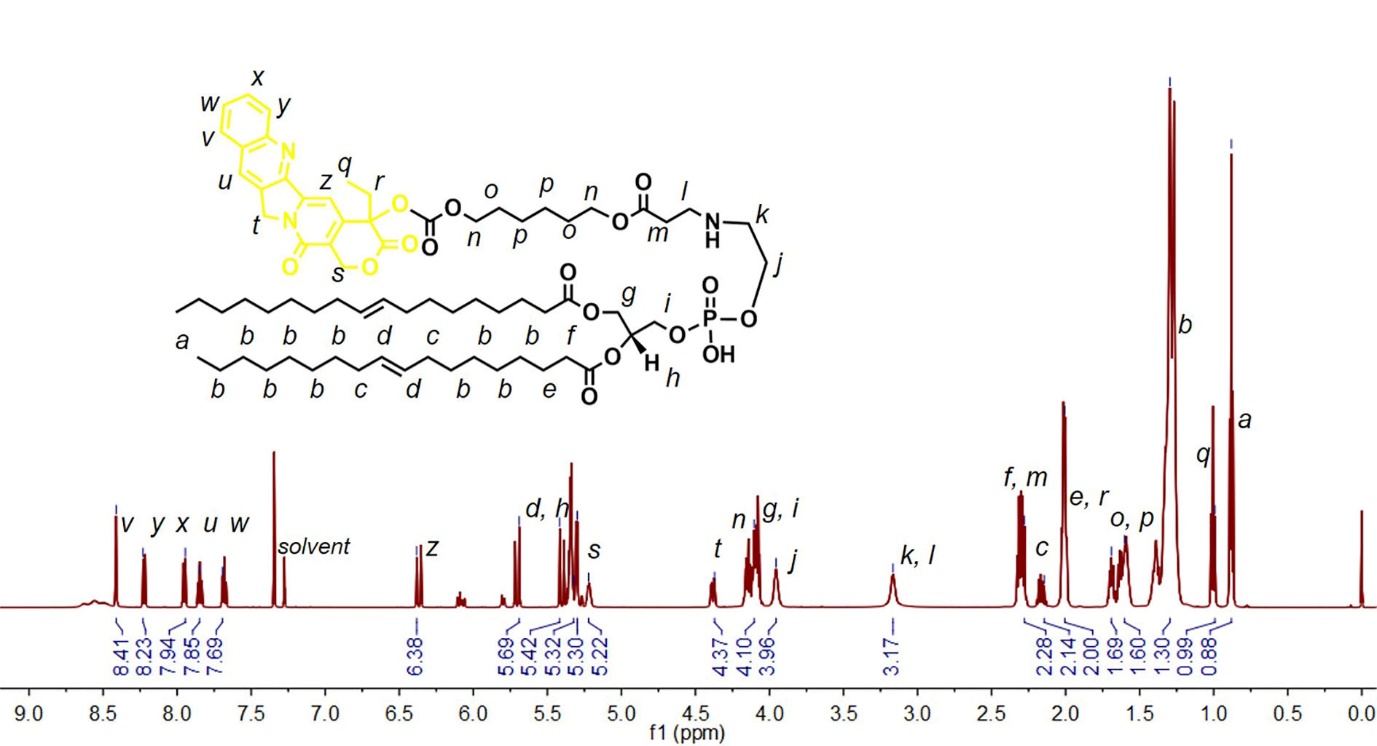
**

**Figure S7.** The ^1^H NMR spectrum of camptothecin-DOPE prodrug conjugate containing hexanediol linker (HCPT) in CDCl_3_ solvent.


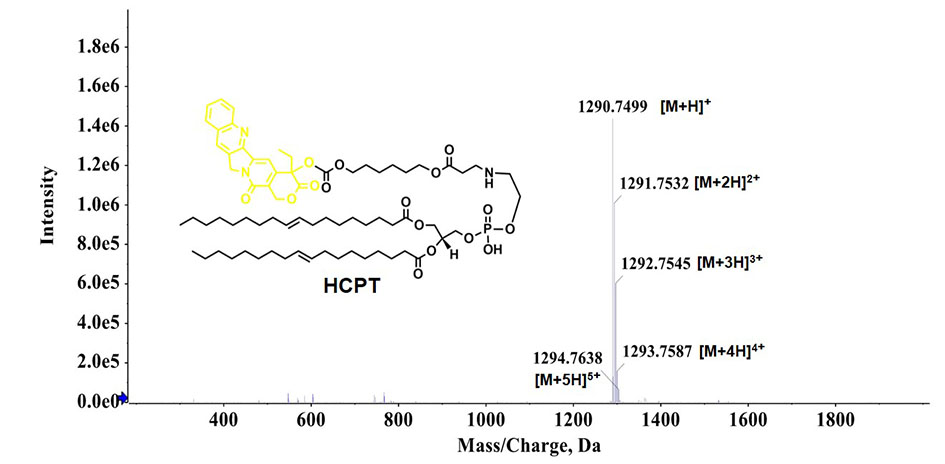


**Figure S8.** The MALDI-TOF-MS spectrum of HCPT.

**Table S1.** The lipid component of liposomes.

| Constituents /Groups | SCPT | Ce6-PEG-DSPE | HSPC/ Chol/  RGD-PEG-DSPE | PEG-DSPE |
| --- | --- | --- | --- | --- |
| CSCPTL | + | + | + | no |
| SCPTL | + | no | + | + |
| CL | no | + | + | no |


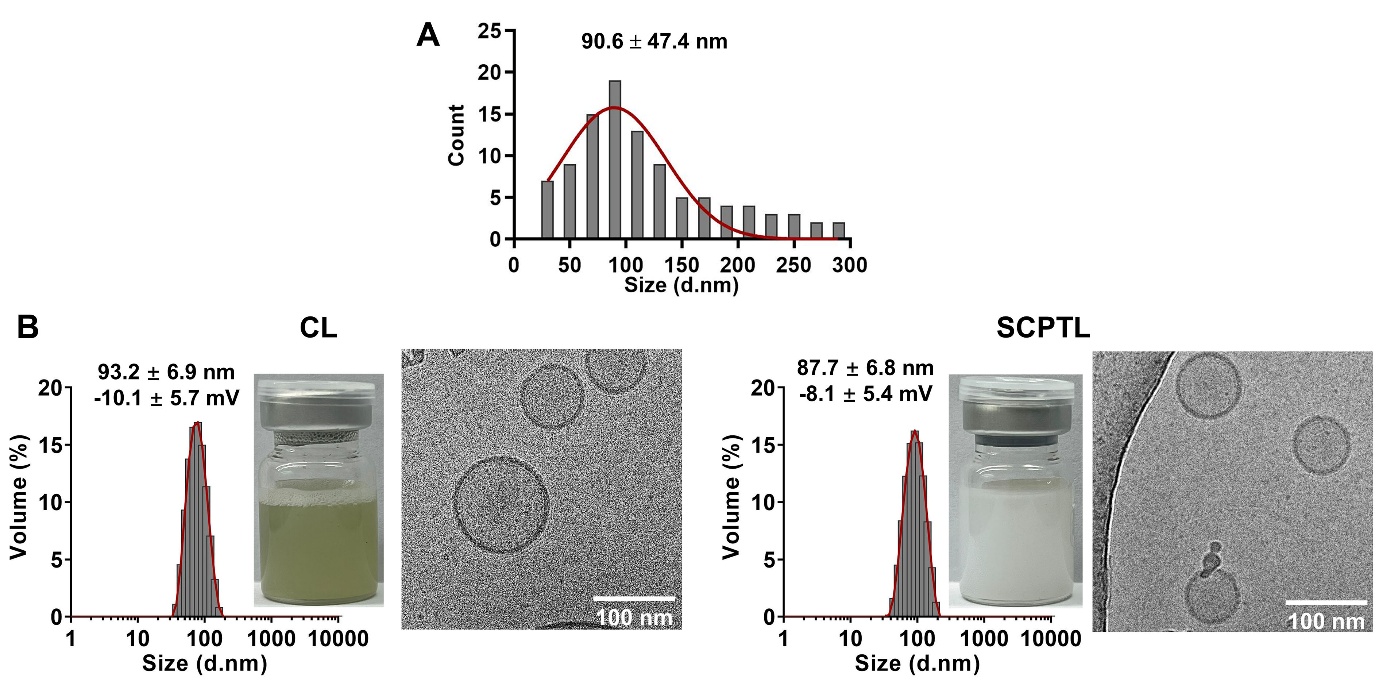


**Figure S9.** (A) The size distribution of CSCPTL in cryo-TEM images measured using the software of Nano Measurer (software version: 1.2.0.5). (B) The macroscopic morphology, size distribution measured by dynamic light scattering, and cryo-TEM images of the control liposomes: CL and SCPTL.

**
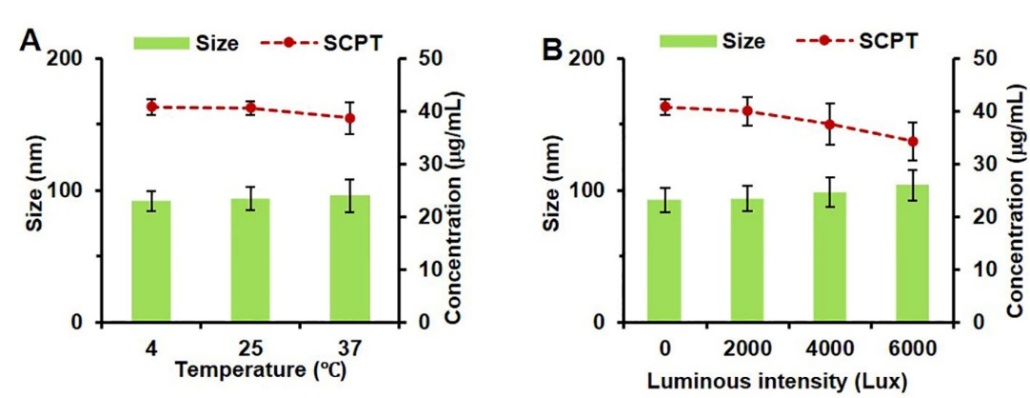
**

**Figure S10.** The stability assay of CSCPTL in the different storage conditions. (A) The changes of particle size and SCTP content in different temperatures of 4°C, 25°C, and 37°C for 48 h in the dark condition. (B) The changes of particle size and SCPT content in different light exposures of 0, 2 000 Lux, 4 000 Lux, and 6 000 Lux at 4°C for 48 h. The experiments were performed using the light stability test chamber equipped with both temperature and light adjuster (Labonce-150CGS-FC, Beijing Labonce Themostatic Technology Company, China). The changes of particle size and SCTP content were respectively tested using dynamic light scattering and HPLC. Data are expressed in mean ± SD (n = 3).


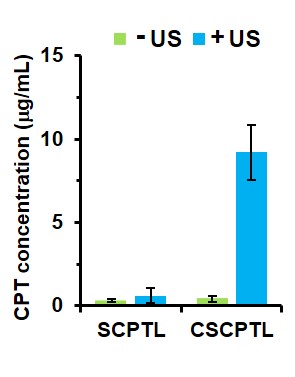


**Figure S11.** Quantitative results of CPT concentration in the liposome solution with or without US irradiation (acoustic intensity: 1 W cm^-2^, frequency: 3 MHz, duty cycle: 50%, duration: 10 min) after 1 h incubation. Data are expressed in mean ± SD (n = 3).

**Figure S12.** The quantitative results of the flow cytometry analysis of the intracellular ROS levels in HepG2 cells in terms of DCFH-DA fluorescence intensity. Data are expressed in mean ± SD (n = 3), unpaired two-tailed Student’s *t*-test, ^**^*P* < 0.01.

**
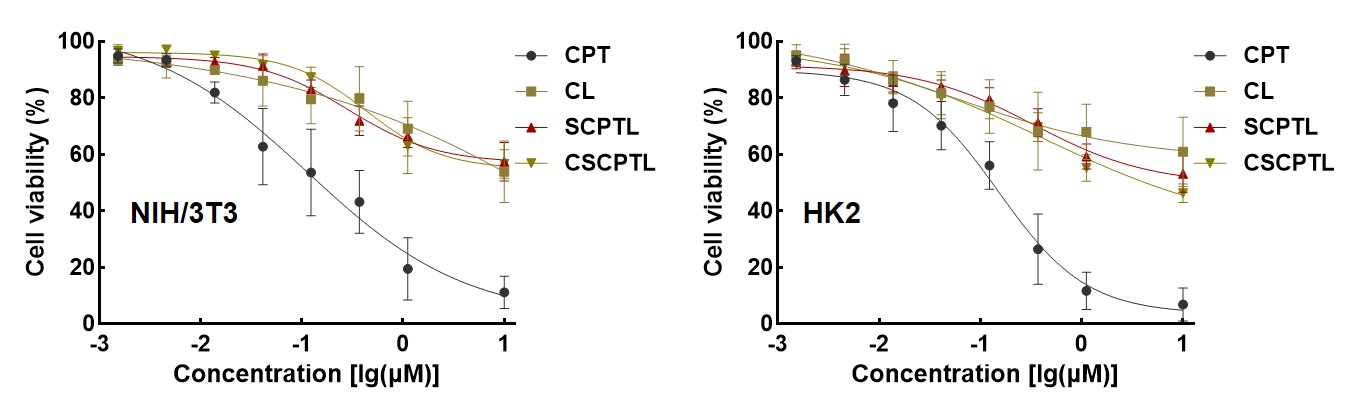
**

**Figure S13.** Cell viability was measured by CCK8 assay after treatment with free CPT and liposomes in NIH/3T3 (a fibroblast cell line) and HK2 (a proximal tubular cell line) without US irradiation under different CPT-equivalent concentrations. Data are expressed in mean ± SD (n = 6).


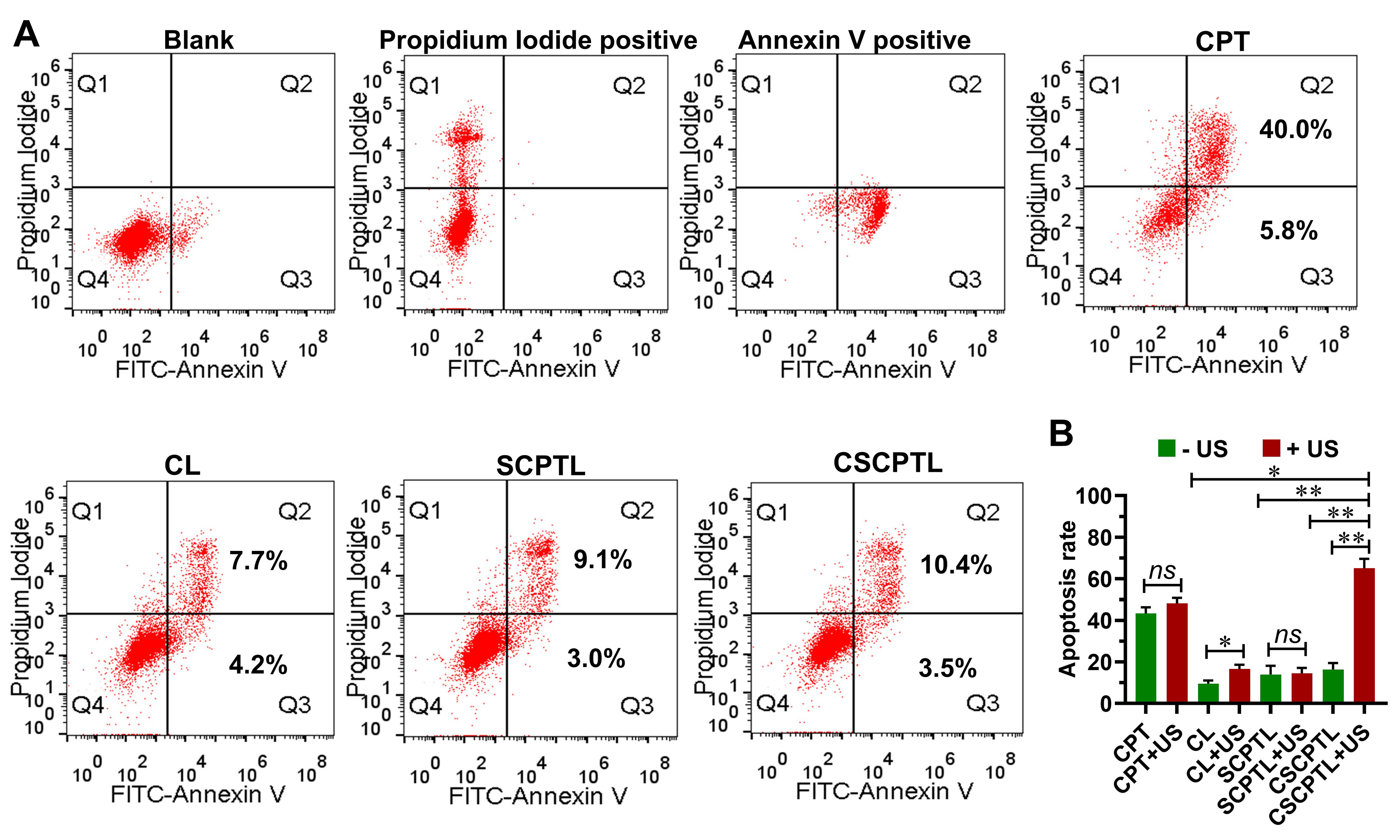


**Figure S14.** (A) Representative flow cytometry analysis of HepG2 cell apoptosis after different treatments without US irradiation at the CPT-equivalent concentration of 0.5 µM. (B) The quantitative analysis of HepG2 cell apoptosis rates after different treatments without or with US irradiation. Data are expressed as mean ± SD (n = 3), significances are determined by unpaired Student’s t-test, ^*^: *P* < 0.05, ^**^: *P* < 0.01, and *ns* means no significant difference.


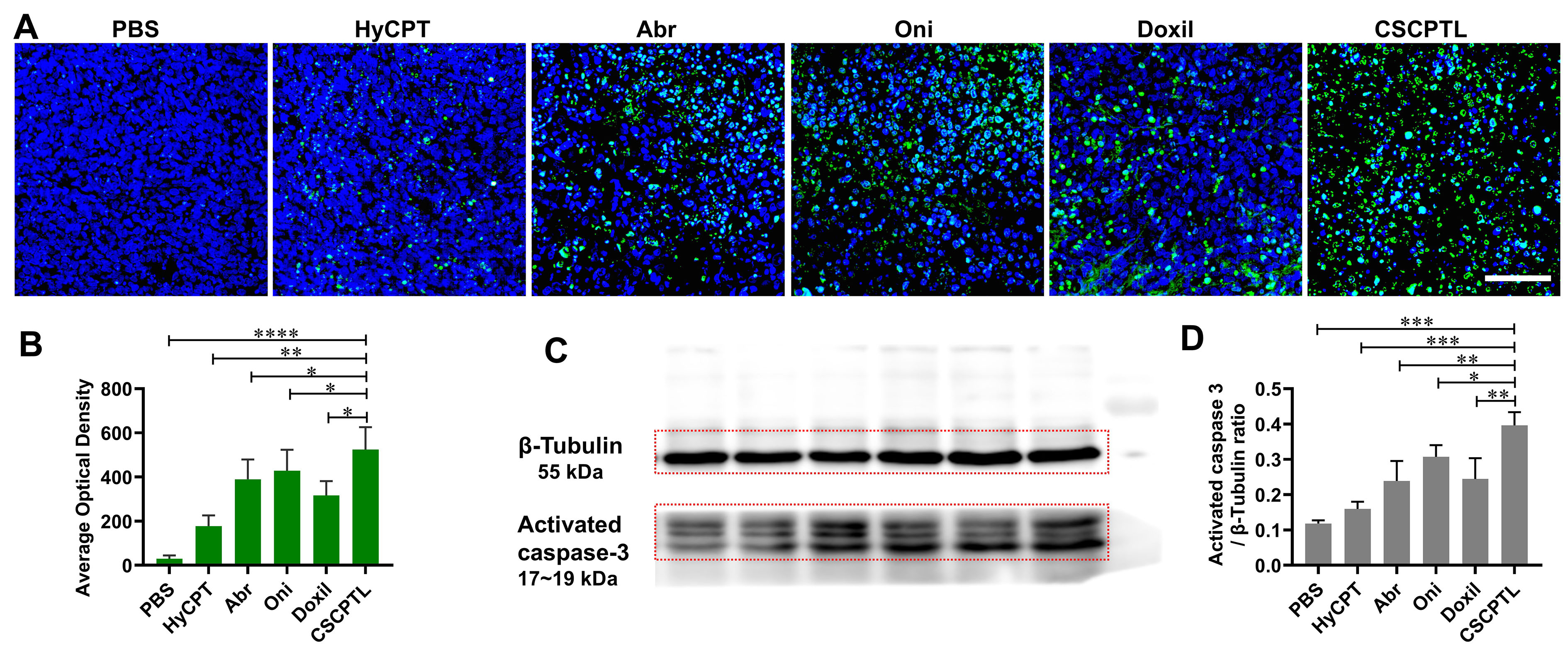


**Figure S15.** (A) Terminal deoxynucleotidyl transferase dUTP nick end labeling (TUNEL) staining of the tumors after different treatments (Scale bar = 100 μm), and (B) the average optical density of TUNEL-positive tumor cells in the tumor sections. Six images in each group were randomly selected and quantitatively analyzed using Image J software (n = 6). (C) The western blotting analysis of the apoptosis-related protein of activated Caspase 3, and (D) its relative quantification from three independent biological replicates (n = 3). The primary antibodies of activated caspase 3 (#25128-1-AP) was purchased from Proteintech Group and used according to the manufacturer's instructions. Data are expressed as mean ± SD, significances are determined by unpaired Student’s t-test, ^*^: *P* < 0.05, ^**^: *P* < 0.01, ^***^: *P* < 0.001, and *ns* means no significant difference.

**References**

[1] Y. Barenholz, *J. Controlled Release* **2012**, 160, 117.

[2] X. Sun, G. Wang, H. Zhang, S. Hu, X. Liu, J. Tang, Y. Shen, *ACS Nano* **2018**, 12, 6179.

[3] G. Wang, Z. Zhou, Z. Zhao, Q. Li, Y. Wu, S. Yan, Y. Shen, P. Huang, *ACS Nano* **2020**, 14, 4890.
